# Theoretical Studies of DNA Microarray Present Potential Molecular and Cellular Interconnectivity of Signaling Pathways in Immune System Dysregulation

**DOI:** 10.3390/genes15040393

**Published:** 2024-03-22

**Authors:** Jon Patrick T. Garcia, Lemmuel L. Tayo

**Affiliations:** 1 School of Chemical, Biological, and Materials Engineering and Sciences, Mapúa University, Manila 1002, Philippines; jptgarcia@mymail.mapua.edu.ph; 2 School of Graduate Studies, Mapúa University, Manila 1002, Philippines; 3 Department of Biology, School of Medicine and Health Sciences, Mapúa University, Makati 1200, Philippines

**Keywords:** autoimmunity, immune dysregulation, hub genes, cytokines, autoantibodies

## Abstract

Autoimmunity is defined as the inability to regulate immunological activities in the body, especially in response to external triggers, leading to the attack of the tissues and organs of the host. Outcomes include the onset of autoimmune diseases whose effects are primarily due to dysregulated immune responses. In past years, there have been cases that show an increased susceptibility to other autoimmune disorders in patients who are already experiencing the same type of disease. Research in this field has started analyzing the potential molecular and cellular causes of this interconnectedness, bearing in mind the possibility of advancing drugs and therapies for the treatment of autoimmunity. With that, this study aimed to determine the correlation of four autoimmune diseases, which are type 1 diabetes (T1D), psoriasis (PSR), systemic sclerosis (SSc), and systemic lupus erythematosus (SLE), by identifying highly preserved co-expressed genes among datasets using WGCNA. Functional annotation was then employed to characterize these sets of genes based on their systemic relationship as a whole to elucidate the biological processes, cellular components, and molecular functions of the pathways they are involved in. Lastly, drug repurposing analysis was performed to screen candidate drugs for repositioning that could regulate the abnormal expression of genes among the diseases. A total of thirteen modules were obtained from the analysis, the majority of which were associated with transcriptional, post-transcriptional, and post-translational modification processes. Also, the evaluation based on KEGG suggested the possible role of T_H17_ differentiation in the simultaneous onset of the four diseases. Furthermore, clomiphene was the top drug candidate for regulating overexpressed hub genes; meanwhile, prilocaine was the top drug for regulating under-expressed hub genes. This study was geared towards utilizing transcriptomics approaches for the assessment of microarray data, which is different from the use of traditional genomic analyses. Such a research design for investigating correlations among autoimmune diseases may be the first of its kind.

## 1. Introduction

Autoimmune diseases are a group of immunity disorders defined by an aberrant immune response in which the body’s tissues, cells, and organs are unintentionally targeted and attacked. In such conditions, the immune system, which is specialized to protect the body from foreign invaders such as pathogens and transplanted material, becomes dysfunctional in distinguishing the identity between “self” and “non-self” molecules. General manifestations include inflammation, skin problems, and fatigue, and the worst is damage to organs and tissues due to misdirection of the immune response [1]. More than 100 autoimmune disorders have been identified, such as type 1 diabetes, psoriasis, systemic sclerosis, and systemic lupus erythematosus. Although the symptoms and origins of the diseases vary, the underlying cause that drives these complications is the same. The etiology of autoimmune diseases is multifactorial, encompassing both genetic influences and environmental susceptibilities. Management may be long-term, since most can lead to chronic health issues and an increased likelihood of developing other autoimmune diseases.

One of the most common autoimmune diseases occurring worldwide is type 1 diabetes (T1D). Also known as insulin-dependent diabetes, T1D is a condition in which the immune system attacks pancreatic β cells that are responsible for producing insulin in the body. The inability to produce insulin results in elevated blood glucose levels, since excess glucose molecules cannot be properly stored. T1D is often characterized by insulitis, inflammation of the islets of Langerhans found in the pancreas, and the presence of β-cell autoantibodies, biomarkers for pancreatic damage caused by T1D [2]. It was found that T1D has a strong correlation with human MHC class II isotypes HLA-DR and HLA-DQ located on chromosome 6. Around 95% of T1D patients express the haplotypes DR3 and DR4, which are associated with haplotypes DQA1*0301-B1*0302 [3].

Moreover, psoriasis (PSR) is a chronic autoimmune skin condition caused by the hyperproliferation of keratinocytes mediated by T cells. The origin of PSR is yet to be fully unraveled; nevertheless, current research suggests that the disease is a manifestation of an overactive immune system, leading to the rapid growth of skin cells [4]. In normal human skin conditions, the ratio of proliferating to nonproliferating keratinocytes is about 50–60%; meanwhile, the same ratio in PSR patients is almost 100% [5]. PSR has several types, which are classified according to which part of the body the rash is observed. PSR rash is characterized by red and silvery-white, scaly plaques on the skin, and in the worst scenarios, it may include the formation of skin lesions. At present, HLA-Cw*0602, an MHC class I molecule, remains the primary genetic indicator of human PSR susceptibility. HLA-Cw*0602 plays a vital role in the presentation of cytoplasmic antigens to CD8+ T cells, which, as previously mentioned, are immune cells known to play a crucial role in the expression of PSR [6].

Systemic sclerosis (SSc) or scleroderma is another untreatable autoimmune disease caused by the overreaction of the immune system. The etiology of the disease is still unknown, but underlying effects involve abnormalities in the microvascular network and connective tissues [7]. Cell-mediated autoimmunity, fibroproliferative vasculopathy, and fibroblast dysfunction are the common manifestations of SSc [8]. Morphea, which is characterized by the swollen-like appearance of the fingers, hands, arms, legs, and face due to the thickening of skin patches, is often observed among SSc patients. Aside from that, complications also affect the blood vessels and other internal organs, including the heart, kidneys, and lungs. A misdirected immune response triggers inflammation of the tissues as if they were damaged, and this causes the body to overproduce collagen in response to the reaction. Just like T1D, the genetic risk factor of SSc is associated with the presence of MHC class II molecules. Haplotypes HLA-DRB1*11-DQB1*0301 are found to be related to the presence of anti-topoisomerase I autoantibodies, while haplotypes HLA-DRB1*01-DQB1*0501 are related to the presence of anti-centromere antibodies [9].

Furthermore, just like other autoimmune diseases, systemic lupus erythematosus (SLE) also occurs when the immune system begins to attack the body’s tissues and organs. Production of autoantibodies is the primary immunologic disturbance commonly occurring among SLE patients. These are generally directed toward “self” molecules found at various cellular sites, including the nucleus, cytoplasm, and cell membrane [10]. Vasculopathy, inflammation of the skin, and immune complex deposition are also pathological findings associated with SLE, and it was found that these manifestations have strong correlations with other autoimmune diseases such as thyroiditis and hemolytic anemia [11]. The haplotypes DR2 and DR3, coming from the HLA class II family, are also linked to SLE. This association accounts for the significant presence of certain autoantibodies, such as anti-Sm, anti-nRNP, and anti-DNA antibodies, during its onset. Aside from that, the genetic susceptibility of the disease is also caused by certain HLA class III molecules, specifically those responsible for activating the production of complement molecules C2 and C4 [12].

Weighted Gene Co-Expression Network Analysis (WGCNA) is a powerful bioinformatics method primarily designed for analyzing and interpreting high-dimensional gene expression data. It is an R package specifically useful in identifying co-expression patterns among genes and exploring the relationships between these and various phenotypes. WGCNA classifies genes into modules or clusters according to how similar their patterns of expression are in different samples [13]. By accounting for the weighted correlations among these genes, this approach makes it possible to identify nuanced co-expression interactions that conventional correlation analyses might have overlooked. The term “weighted” in WGCNA alludes to the fact that it uses varying weights to indicate distinct pairwise gene correlations, downweighing weaker correlations while emphasizing stronger correlations [14]. Functionally related genes are those that have a similar pattern of expression and are closely associated with one another within a module. A more comprehensive understanding of the biological pathways and mechanisms that underlie the observed patterns of gene expression can be obtained through this arrangement. The ability of WGCNA to identify gene modules that are co-expressed and linked to particular traits is one advantage in studying systems biology [15]. Modules that are biologically related to a phenotype can be recognized by comparing module eigengenes, which are summary profiles of module expression. This may result in the discovery of putative biomarkers and therapeutic targets or provide a better understanding of molecular processes underlying a certain disease.

In this study, we used WGCNA in identifying gene expression patterns across four autoimmune diseases: T1D, PSR, SSc, and SLE. Recent studies have shown the possible coexistence of these conditions, and medical records have presented actual cases where these were manifested among patients with aggravated autoimmune conditions. Moreover, we determined highly preserved co-expressed genes among the datasets for functional annotation to determine common pathways that could enlighten the underlying genetic factors that explain the interconnectedness of the four diseases, answering the question as to why patients with an already-existing autoimmune disease a have high risk of developing another autoimmune disorder. We also highlight the hub genes present among the modules that play a central role in the common manifestations of the diseases. Drug repurposing analysis was also undertaken to present possible drugs that may regulate them. This study tackles the problem at the transcriptomics level, which is different from most medical research, whose objectives are geared towards genomic assessment. The use of WGCNA as a method for gene expression analyses of autoimmune diseases may be a novel approach in terms of opening opportunities to address gaps in drug development and therapy.

## 2. Materials and Methods

### 2.1. Evaluation of Autoimmune Disease Candidates

Microarray data of the four diseases were obtained from the National Center for Biotechnology Information—Gene Expression Omnibus (NCBI-GEO) [https://www.ncbi.nlm.nih.gov/geo/ (accessed on 10 January 2024)]. It is an online public repository of genomics data, including multifarious microarray and high-throughput sequencing data, submitted by various research institutes all over the world. The GEO datasets used in the study were evaluated based on the following set of criteria for uniformity: (1) the samples must have been obtained from humans or *Homo sapiens*, (2) the sample source must have been extracted from blood, (3) the experiment type must be ‘expression profiling by array’, (4) the platform used must be Affymetrix Human Genome U133 Plus 2.0 Array, (5) the number of positive samples must be ≥20, and (6) the number of negative controls must be ≥20. Only those GEO datasets that complied with the mentioned parameters and that had available TAR (of CEL) supplementary files were chosen for the study. A summary of the GEO datasets used in the study is shown in Table 1.

The chosen T1D dataset (GSE35725) contains 44 positive samples and 44 negative controls taken from a study that sought to identify expressed genes in the peripheral blood mononuclear cells of T1D patients [16]. The chosen PSR dataset (GSE55201) contains 44 positive samples and 30 negative controls taken from a study that characterized transcriptional changes in the blood of PSR patients after IL-17 treatment [17]. The chosen SSc dataset (GSE65336) contains 29 positive samples and 29 negative controls taken from a study that aimed to evaluate the effects of anifrolumab and type 1 IFN in the regulation of scleroderma [18]. Lastly, the chosen SLE dataset (GSE61635) contains 64 positive samples and 30 negative controls taken from a study that assessed gene expression profiles between SLE patients and healthy donors, focusing on cytokines central to B-cell activation and differentiation [19].

### 2.2. Preliminary Screening through Differential Expression Analysis

To show the possible coexistence of the four diseases, a preliminary analysis was performed by determining the presence of overlapping differentially expressed genes (DEGs) among the four datasets through differential expression analysis (DEA) via GEO2R accessed from the NCBI-GEO web server. GEO2R [https://www.ncbi.nlm.nih.gov/geo/geo2r/ (accessed on 10 January 2024)] is a bioinformatics tool that compares two or more groups of microarray samples to determine genes that are highly expressed or regulated across varying experimental conditions. To reduce false positives by adjusting the *p*-values of the samples, Benjamini and Hochberg’s false-discovery rate was employed [20]. Meanwhile, the limma precision weights or vooma were used to calculate the mean–variance relationship [21]. Force normalization was applied for the log transformation and identical value distribution of the samples. Only those genes that passed the adjusted *p*-value cutoff of <0.05 were retained and grouped either as upregulated, for a log_2_fold (FC) score of 0, or downregulated, for an FC score < 0. Both groups obtained from the four datasets were then sent to the Bioinformatics & Evolutionary Genomics web server [https://bioinformatics.psb.ugent.be/webtools/Venn/ (accessed on 10 January 2024)] to identify overlapping DEGs across the diseases using a Venn diagram.

### 2.3. Weighted Gene Co-Expression Analysis (WGCNA) of Datasets

#### 2.3.1. Pre-Processing of Datasets

Using Bioconductor v3.18 [https://www.bioconductor.org/ (accessed on 10 January 2024)], which is an open software equipped with features for computational biology studies, each dataset was loaded into the R program v4.3.2 [https://cran.r-project.org/bin/windows/base/ (downloaded on 24 January 2024)] for pre-processing. Along with that, the “affy” and “biomaRt” packages were also installed, the former of which is intended for oligonucleotide array analysis and the latter of which is used for the easy and uniform retrieval of large data without the need for complex database schemas [22]. Each raw data point obtained from the datasets was normalized using the Robust Microarray Analysis (RMA) function in the program. Then, the expression data of the four datasets were extracted, unnecessary extensions found in the samples were eliminated, and control probes were removed. After which, the mean and variance of the expression data were calculated and the 20% percentile of each was determined. The expression data were filtered such that only those genes that obtained a mean and variance higher than the 20% cutoff were retained. IDs of common probes among the expression data were converted to gene symbols, and those entries without gene symbols were removed. Finally, the expression data were subjected to log-2 transformation using the “goodSamplesGenesMS” function to filter out genes that contained missing values. Initial weighted gene co-expression networks were also generated with the aid of scattered plot diagrams to check the comparability of the datasets before modularization. Each dataset was compared to the others, with plots showing the following dataset comparisons: T1D vs. PSR, T1D vs. SSc, T1D vs. SLE, PSR vs. SSc, PSR vs. SLE, and SSc vs. SLE.

#### 2.3.2. Quantitative Determination of Reference Dataset

The soft-thresholding power (*β*) was determined by plotting the network topology of each dataset, as well as the mean and median connectivity fits to confirm the result, using the “pickSoftThreshold” function from the WGCNA package. The value of *β* at which the plots begin to flatten out was chosen as the soft-thresholding power in constructing the respective scale-free topologies of the datasets. To further evaluate the *β* of choice, scatter plots were generated, and the correlation coefficient of each was calculated. The dataset with the highest fit at a particular value of *β* and the highest correlation coefficient was used as the reference dataset for succeeding analyses.

#### 2.3.3. Formulation of Co-Expression Networks

The chosen value of *β* was then utilized to calculate the adjacency matrices of the reference dataset using the “adjacency” function through a “signed” network type via Pearson’s correlation. After this, the results were subjected to topological overlap measure (TOM) dissimilarity using the “1-TOMsimilarity” function through a “signed” TOM type. This was to perform hierarchical clustering of the genes in the reference dataset to identify co-expression networks or functional modules within the clusters. A dendrogram was then plotted to show the tips of the branches, which are associated with those highly correlated genes found in the reference dataset. These tips denote the clusters from which modules were identified. Aside from that, a dynamic tree-cutting algorithm was also utilized using the hybrid tree cut. A deep-split parameter ranging from 0 to 3 was evaluated to control the sensitivity of the algorithm, as well as to better visualize the distribution of the modules per deep split.

#### 2.3.4. Calculation of Module Preservation and Membership

Using the “modulepreservation” function from the WGCNA package through a “signed” network type, the weighted gene co-expression network preservation of the reference dataset was calculated relative to the other three datasets. The number of permutations was set to 100, and the maximum module size was set to 10,658, which is the total number of genes obtained from the grey module. Those modules that were found to be highly preserved across the datasets, that is, modules that obtained a *z*-score >10 in the other three datasets, were used for succeeding analyses. Afterward, the modules were further processed by calculating the eigengene-based connectivity (kME) of each gene using the “moduleEigengenes” function from the WGCNA package to quantify their respective connectivity among the other genes within the module. The module membership of each dataset was then programmed by correlating the eigengene and expression profile of each gene, and these were ranked from highest to lowest within the modules. Scatter plot diagrams under a set *p*-value < 0.05 were drawn to visualize these correlations.

### 2.4. Functional Annotation of Pathway Enrichment of Modules

Each module was sent to the Database for Annotation, Visualization, and Integrated Discovery (DAVID) web server for functional annotation clustering [23,24]. DAVID [https://david.ncifcrf.gov/ (accessed on 10 January 2024)] is a bioinformatics tool provided by the National Institute of Health (NIH) that contains sophisticated functional annotation programs for better understanding the biological mechanisms underlying sets of genes. Due to the limitation of the web server, only the top 3000 genes based on the ranking from the module membership were used for this analysis. Biological process (BP), cellular component (CC), and molecular function (MF) under the gene ontology classification and KEGG under the pathway classification were the selected annotations for clustering the genes in each module, with stringency set to “medium”. Only the terms with the highest enrichment scores and *p*-values < 0.05 were recorded for each annotation from every module and were sent to the SRplot web server [http://www.bioinformatics.com.cn/srplot (accessed on 10 January 2024)] for enrichment bubble visualization.

### 2.5. Identification of Hub Genes from Protein–Protein Interaction (PPI) Networks

The stringApp v2.0.2 [https://apps.cytoscape.org/apps/stringapp (downloaded on 10 January 2024)] and cytoHubba v0.1 [https://apps.cytoscape.org/apps/cytohubba (downloaded on 10 January 2024)] applications were installed in the Cytoscape v3.10.1 [https://cytoscape.org/index.html (downloaded on 10 January 2024)] software prior to network analysis. StringApp is intended for augmentation of PPI networks sourced from the Search Tool for Recurring Instances of Neighbouring Genes (STRING) v12.0 [https://string-db.org/ (accessed on 10 January 2024)] web database, while cytoHubba is specialized for predicting nodes and subnetworks in a PPI network to explore hub genes based on various topological algorithms. Each module was uploaded to the Cytoscape software under a minimum interaction score of 0.7, which is equivalent to a high confidence level, and a large network of interactions was generated for each module. After which, the constructed network was submitted to cytoHubba to identify the central hub genes for every module. The following three topological algorithms were utilized: maximum neighborhood component (MNC), degree, and closeness. The top 50 hub genes were obtained for each algorithm, and using the same Venn diagram online tool, the overlapping hub genes in all three algorithms were identified and analyzed further. A PPI network was drawn from Cytoscape to calculate the connectivity among these hub genes.

### 2.6. Drug Repurposing Analysis of Hub Genes

All the hub genes were sent again to GEO2R for DEA, where they were classified either as upregulated or downregulated. The two groups were then fed to the Drug Repurposing Encyclopedia (DRE) web server, where they were subjected to drug repurposing. The DRE [https://www.drugrep.org/drugrepurposinganalysis (accessed on 10 January 2024)] is an online drug discovery platform that is referenced from four large drug databases intended for drug repurposing analyses, screening over a total of 4690 consensus drugs from 20 studied organisms [25]. The DEGs were treated as gene signatures and inputted into the DRE. Drugs whose status is either experimental or withdrawn were excluded. Only the top 5 drugs with the most negative Tau scores, FDR values < 0.05, and that are approved for human use were recorded.

## 3. Results

### 3.1. Preliminary Screening through Differential Expression Analysis

All four datasets were sent to GEO2R for DEA, in which DEGs were identified by comparing changes in the expression of genes between the positive samples and the negative controls. The DEGs from each dataset were grouped either as upregulated or downregulated based on a set adjusted *p*-value and FC thresholds. The T1D dataset has 420 upregulated DEGs and 274 downregulated DEGs, the PSR dataset has 332 upregulated DEGs and 318 downregulated DEGs, the SSc dataset has 612 upregulated DEGs and 776 downregulated DEGs, and the SLE dataset has 761 upregulated DEGs and 366 downregulated DEGs.

**Figure 1:**
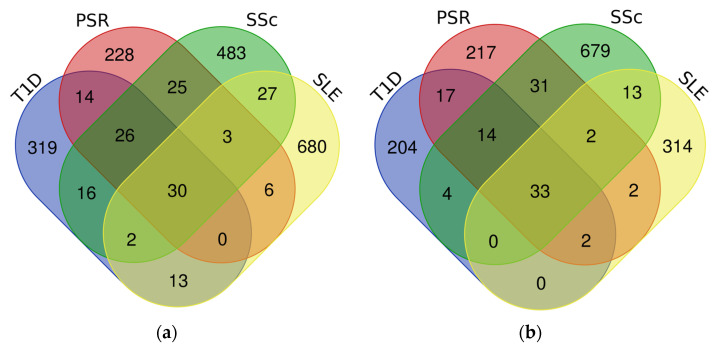
Venn diagram of the upregulated and downregulated groups in determining the overlapping DEGs among the four datasets: (**a**) upregulated DEGs; (**b**) downregulated DEGs.

After this, the DEGs were compared across the datasets to determine or identify overlapping DEGs within the two groups using a Venn diagram, as shown in Figure 1. This further confirms the possible genetic coexistence of the four diseases, with 30 common upregulated DEGs and 33 common downregulated DEGs.

### 3.2. Normalization and Filtering of Datasets

Using the R program, the datasets were normalized via the RMA method, and the expression data from the results were extracted. Furthermore, unnecessary extensions and control probes were removed. The remaining genes that obtained a mean and variance greater than the 20% cutoff were converted into gene symbols, and those without gene symbols were eliminated. Lastly, log-2 transformation was performed, and those with missing values from the expression data were filtered out. After a series of pre-processing steps, a total of 20,350 genes remained. Moreover, Figure S1 shows the general network properties of the datasets as initial constructs for comparability testing. All the obtained ranked expression scattered plot diagrams exhibited positive correlation, which suggests the presence of co-expression genes among the datasets. The SSc vs. SLE comparison attained the highest correlation coefficient of 0.87, while the PSR vs. SLE comparison attained the lowest correlation coefficient of 0.67. Aside from the presence of common DEGs, these results further support the viability of the samples to be used for WGCNA.

### 3.3. Approximation of Scale-Free Networks

**Figure 2:**
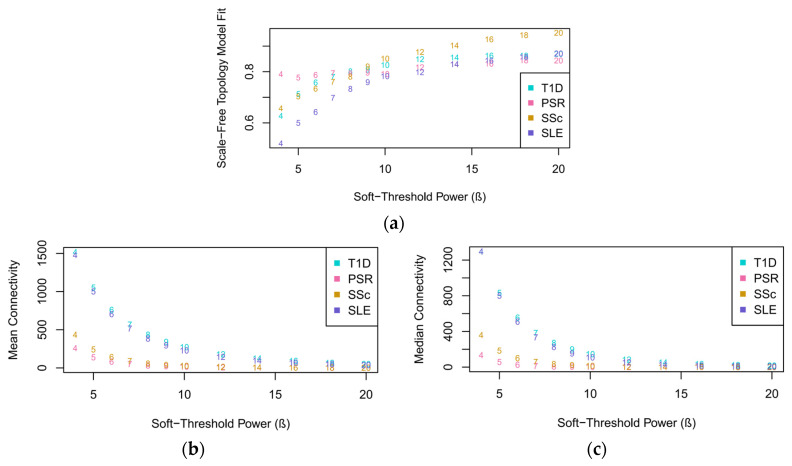
Network index plots for determining the soft-thresholding power approximated from the four datasets: (**a**) scale-free topology model fit; (**b**) mean connectivity; (**c**) median connectivity. The mean and median connectivity plots measure the average and intermediate number of connections per gene, respectively, in the network construct, supporting the overall interconnectedness and suitability of the chosen β.

A scale-free topology model fit, including the mean and median connectivity fits, of the datasets was generated to determine the appropriate soft-thresholding power (*β*) in constructing the network model. As observed in Figure 2a, the datasets started to flatten out at a *β* value of 20; thus, it was chosen for calculating the adjacency matrices. At the lowest *β* value, SLE had the lowest scale-free topology fit, while PSR had the highest fit. However, only T1D, SSc, and SLE had a steady exponential increase in slope with an increasing *β*. PSR showed inconsistencies in its plot, as characterized by the variations in its slope with *β* values in the range of 5–12, but eventually flattened out at 20. At this chosen *β*, PSR attained the lowest scale-free topology fit, while SSc obtained the highest fit.

The choice of 20 as the *β* value for constructing the network model was further confirmed by the plots obtained from the mean and median connectivity fits. As seen in Figure 2b,c, the datasets exhibited mean and median connectivity at a *β* value of 20, although there was greater connectivity in the median fit than the mean fit, as shown by the difference in the overlapping of the plots between the two figures. Overlapping started at a *β* value of 18 in the mean connectivity, while it started at 14 in the median connectivity.

**Figure 3:**
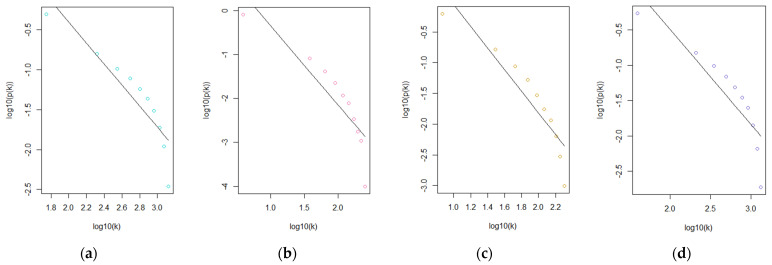
Straight-line relationship of the four datasets using *β* = 20 as the soft-thresholding power: (**a**) T1D; (**b**) PSR; (**c**) SSc; (**d**) SLE.

Subsequently, scatter plots of the four datasets, as shown in Figure 3, were drawn, and their respective correlation coefficients were calculated to provide another quantitative basis for choosing the reference dataset. SSc had the highest correlation coefficient of 0.83, followed by T1D with 0.8 and PSR and SLE with 0.76.

Based on the scale-free topology model fit and straight-line relationship results, SSc was chosen as the reference dataset for modeling the co-expression networks. However, aside from solely relying on the computations in choosing the reference dataset, it has been reported that among the four diseases, SSc commonly coexists with either T1D, PSR, or SLE in patients with an aggravated autoimmune condition.

One study reported the coexistence of T1D and SSc when a 14-year-old T1D patient was found to have developed SSc upon the diagnosis of cheiroarthropathy, which is characterized by the thickening of dorsal and palmar surfaces of the skin due to long-standing uncontrolled diabetes [26]. Positive autoantibodies, aberrant nailfold capillaroscopy with scleroderma patterns, interstitial lung disease, and cardiac involvement were all detected after a thorough work-up of the case, which confirmed the diagnosis of SSc in the patient [27,28,29]. On the other hand, T_H17_ cells and IL-17 molecules were found to be involved in the pathogenesis of both PSR and SSc [30,31]. In SSc, B cells aid CD4+ T cells to develop into T_H17_ cells [32], and the T_H17_-cell count suggests how the severity of SSc can prime the development of PSR [33]. IL-17 produced by T_H17_ cells plays a mechanistic role in collagen overproduction and fibroblast proliferation, which are manifestations in both SSc and PSR, elucidating the significant impact of this pathway on their coexistence [34]. Lastly, SSc and SLE are comparatively defined as multisystem autoimmune connective tissue diseases [35]. There are many similarities between SLE and SSc, including autoantibodies against nuclear antigens and, in certain cases, similar clinical characteristics. There is growing evidence supporting the correlations between SSc and SLE at the gene level, such as the presence of IRF5 [36,37,38] and PTPN22 [39,40,41] involved in their pathways. These genes are linked to high serum levels of IFN-α in SLE patients [42,43], further confirming its possible and simultaneous occurrence with SSc.

### 3.4. Identification of Co-Expressed Modules

Choosing a reference dataset and projecting the eigengenes of other datasets against the reference is one meta-analytical approach in WGCNA [44,45,46,47]. The number of samples, the scale-free network, and the TOM-based gene dendrogram of the reference affect the robustness of the calculated network [47,48]. From the network construct modeled from the calculated adjacency matrices of the SSc dataset, TOM dissimilarity and a dynamic tree-cutting algorithm were applied to determine highly correlated genes from where the modules were identified. A dendrogram was drawn to show the tips that are associated with those highly correlated genes in the SSc dataset. On the other hand, a deep-split parameter of 0–3 was used to visualize possible modules that can be obtained from these correlated genes. Figure S2 displays the dendrogram of gene clustering in the SSc dataset, where it can be observed that the tips from the dendrogram could be associated with particular modules in a certain deep-split parameter generated from the dynamic tree-cutting algorithm.

**Figure 4:**
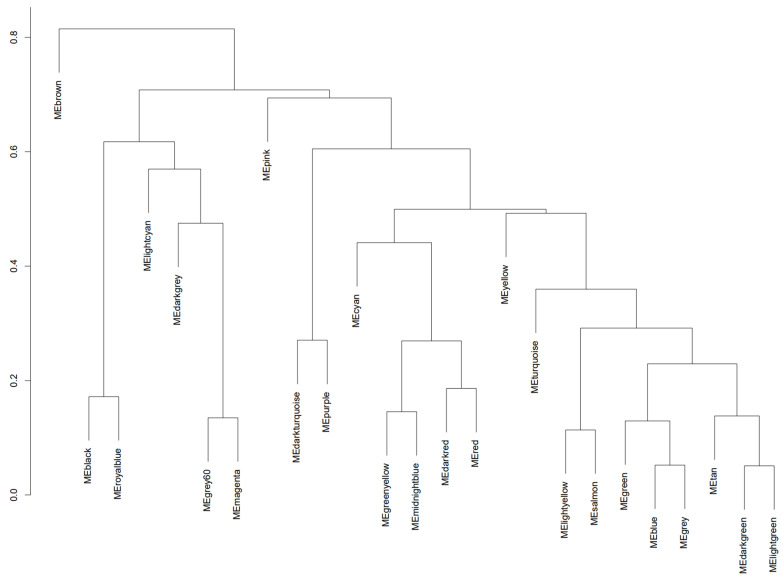
Dendrogram of the reference dataset containing co-expressed genes clustered into networks that are represented by modules. Modules coming from the same branch have relatively similar expression patterns but may have different biological manifestations.

Using a deep-split parameter of 1 to obtain larger modules while maintaining efficiency in the sensitivity of the algorithm [49], we obtained 25 co-expression modules that are represented by various colors, as seen in Figure 4. The turquoise module contains 1476 genes, the blue module contains 1356 genes, the brown module contains 951 genes, the yellow module contains 761 genes, the green module contains 730 genes, the red module contains 619 genes, the black module contains 523 genes, the pink module contains 358 genes, the magenta module contains 327 genes, the purple module contains 285 genes, the green–yellow module contains 268 genes, the tan module contains 248 genes, the cyan module contains 245 genes, the salmon module contains 245 genes, the light-cyan module contains 229 genes, the midnight-blue module contains 229 genes, the grey60 module contains 209 genes, the light-green module contains 200 genes, the light-yellow module contains 183 genes, the royal-blue module contains 164 genes, the dark-red module contains 128 genes, the dark-green module contains 116 genes, the gold module contains 100 genes, the dark-turquoise module contains 78 genes, and the dark-grey module contains 44 genes.

### 3.5. Module Preservation and Module Membership

The modules obtained from SSc were quantitatively compared to the T1D, PSR, and SLE datasets by computing their respective *z*-scores to measure the density and connectivity of the modules. Those that obtained a *z*-score < 10 across the three datasets were considered highly preserved [50]. Differences in the *z*-scores of the modules in the three datasets suggest that the modeled co-expression networks showed variations in conservation among the diseases. Some of the modules may be highly preserved in one disease and may be poorly preserved in another, and these instances could be due to genes that are only specific for a certain disease. Those modules that passed the *z*-score threshold in all three datasets were then subjected to module membership.

**Figure 5:**
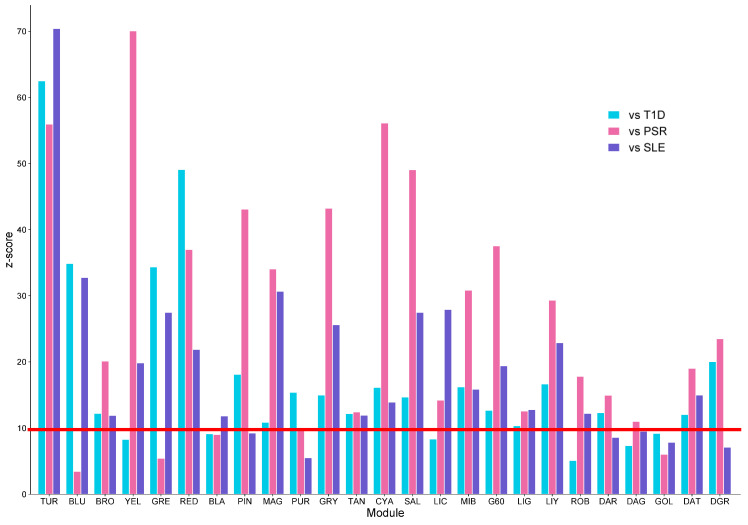
Bar plot results of the module preservation analysis across the three datasets against the reference. Once all three bars reach the red line, the module is said to contain genes that are highly preserved among the diseases. TUR—turquoise; BLU—blue; BRO—brown; YEL—yellow; GRE—green; RED—red; BLA—black; PIN—pink; MAG—magenta; PUR—purple; GRY—green yellow; TAN—tan; CYA—cyan; SAL—salmon; LIC—light cyan; MIB—midnight blue; G60—grey60; LIG—light green; LIY—light yellow; ROB—royal blue; DAR—dark red; DAG—dark green; GOL—gold; DAT—dark turquoise; DGR—dark grey.

Figure 5 shows the preservation analysis of the 25 modules from the SSc network. Only 13 modules were highly preserved across the three datasets, as follows: turquoise, brown, red, magenta, green yellow, tan, cyan, salmon, midnight blue, grey60, light green, light yellow, and dark turquoise. The red line highlights the *z*-score threshold equal to 10.

The connectivity of each gene from the remaining 13 modules was calculated using eigengene-based connectivity (kME). The eigengene of the module, which serves as the first principal component of that module, was compared to the expression profile of the gene, and their correlation was calculated [44,50]. Based on the obtained kME values, the genes were ranked in each module from highest to lowest. Those top genes are deemed to be highly connected—in other words, the genes that are greatly functional within the co-expressed network—which ascertains the preservation of these genes across the datasets. With that, this elucidates the possible key roles or underlying biological mechanisms of the modules based on the ranking of these genes.

### 3.6. Functional Annotation of Hub Genes

**Figure 6:**
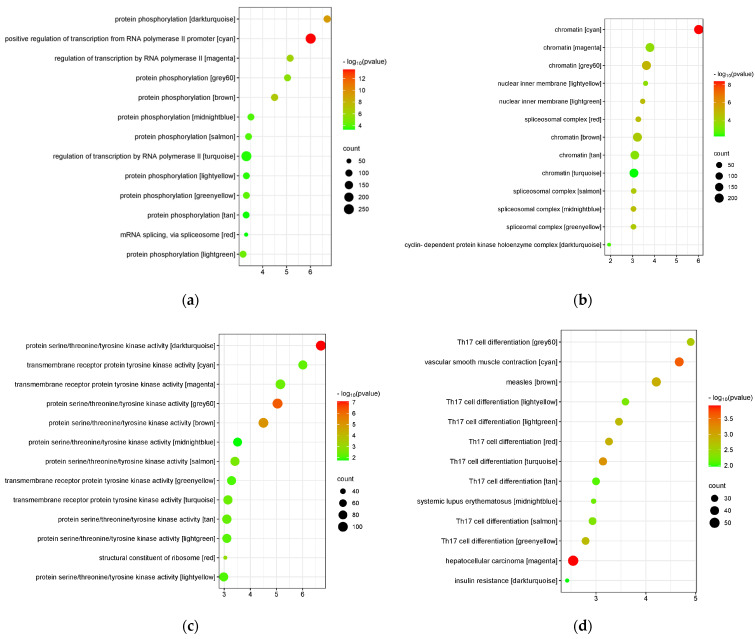
Bubble plot of the top terms from each annotation based on the cluster with the highest enrichment score: (**a**) biological process; (**b**) cellular component; (**c**) molecular function; (**d**) KEGG pathway.

The modules were sent to the DAVID web server for functional annotation. They were clustered based on BP, CC, MF, and KEGG annotations. Due to an overwhelming amount of information obtained from the analysis, only the terms with the highest enrichment scores and *p*-values < 0.05 for each annotation were sent to the SRplot web server for bubble plot visualization. Figure 6 shows the bubble plots of the BP, CC, MF, and KEGG annotations of the modules based on their enrichment scores and *p*-values. These top terms suggest the main biological processes, cellular components, and molecular functions involved in identifying the biological mechanism of the module, as well as the most similar KEGG pathway from which the module could be highly associated with. The top five annotations from each module are listed in detail in Table S1.

### 3.7. Identification of Hub Genes from Protein–Protein Interaction (PPI) Networks

Using the STRING database, all the genes from each module were sent to Cytoscape to predict their respective PPI networks. MNC, degree, and closeness algorithms were utilized from cytoHubba to augment the hub genes from each module. Each algorithm provides one PPI network; thus, three PPI networks were generated. To obtain a better understanding of the biological mechanism underlying each module, the top 50 hub genes calculated using the algorithms were obtained, and these were compared with each other to determine the hub genes greatly preserved or overlapping among the PPI networks. The overlapping hub genes are listed in Table S2.

Hub genes are frequently at the center of regulatory pathways and networks that control immune responses. Researchers can discover important participants in the dysregulated immune system and obtain a deeper comprehension of the molecular underpinnings of autoimmune disorders by finding hub genes linked to these conditions. These could also be used as autoimmune disease biomarkers such that variations in these genes’ expression levels may be a sign of how a disease is developing or how a treatment is working. Biomarkers can help with prognosis, diagnosis, and tracking of how well therapeutic interventions are working. Several genes, proteins, and signaling pathways interact intricately in autoimmune disorders. The network perspective reveals how these various components cooperate or are dysregulated within the framework of the immune system, aiding in further understanding the complexity of these interactions. With that, a PPI network was constructed from Cytoscape to visualize the interrelationships of the overlapping hub genes from each module, as shown in Figure S3.

### 3.8. Drug Repurposing Analysis of Hub Genes

The hub genes were again sent to GEO2R for DEA, where they were classified as upregulated or downregulated. The upregulated hub genes were CD8A, CCL5, TP53, MED1, CD4, SYK, BCL2, PRKCA, GNB1, HSP90AB1, PIK3R1, SMAD3, TOP2A, FYN, CDK2, MRPL3, RPL35, RPS5, RPS24, CD40, and IMP3. Meanwhile, the downregulated genes were CD44, BRCA1, TLR4, ITGAM, STAT1, MYC, JUN, CASP3, CCNA2, FOS, MAPK1, CXCR4, CCL2, MAPK14, TLR2, CXCL8, TGFB1, IL1B, ICAM1, MAPK3, APOE, MMP9, PTPRC, JAK2, GSK3B, CTNNB1, EZH2, DDX58, and PTEN. Then, the groups were sent to the DRA web server for drug repurposing. Clomiphene was the top-ranked drug candidate for the upregulated group, and prilocaine was the top-ranked drug candidate for the downregulated group. Table 2 summarizes the top five drug candidates obtained from DRE in regulating the abnormal expression of hub genes.

## 4. Discussion

### 4.1. Role of Immune System Dysregulation in T1D, PSR, SSc, and SLE

The respective pathogeneses of T1D, PSR, SSc, and SLE have not been fully elucidated yet, but recent studies have shed light on the role of the immune system in activating the onset and, eventually, the aggravation of these diseases. Both environmental and genetic cues are found to be possible causes of the failure of molecular and cellular mechanisms in the body to follow regulatory restrictions, which result in such autoimmunity. This dysregulation of the immune response triggers a cascade of immunological effects that attack or kill certain functional cells that later manifest as various autoimmune diseases. Table 3 summarizes the effect of immune dysregulation on the stimulation of unrestricted pathways responsible for triggering the diseases.

### 4.2. Gene Co-Expression Modules among T1D, PSR, SSc, and SLE Datasets

According to the functional annotation and pathway enrichment analysis through DAVID, the majority of the modules were associated with T_H17_ differentiation. Protein phosphorylation, chromatin, and protein serine, threonine, or tyrosine kinase activity involvement were also identified among most of the modules. These results suggest the molecular and cellular mechanisms of the pathways that the four diseases may have in common.

#### 4.2.1. Implication of Transcriptional Polymorphisms for the Pathophysiology of Autoimmunity

**Figure 7:**
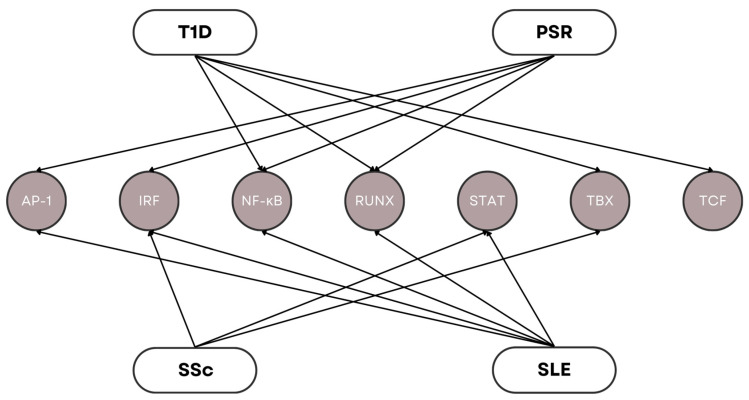
Gene families of transcription factors involved in the pathophysiology of T1D, PSR, SSc, and SLE.

Genetic triggers that lead to autoimmunity greatly impact the long-term phenotypic manifestations of autoimmune diseases [63,64]. Dysfunction of components in the transcription process often results in abnormal expression of certain genes, which can affect the highly regulated stages of the immune response [65]. There has been a growing interest in the study of transcription factors as main regulators in the development and function of immune effector cells. These effector cells interact with other immune cells, such as T cells, B cells, macrophages, and neutrophils, to carry out specific immune responses. Recent genetic studies have linked transcription factors with the pathogenesis of autoimmune diseases. Figure 7 illustrates the gene families of various transcription factors associated with T1D, PSR, SSc, and SLE. All these genes were found to be present among the modules, indicating the prominence of transcription dysregulation in the onset of these four diseases.

SNPs have been discovered to affect transcription binding sites in certain autoimmune disorders [66]. The occurrence of SNPs in DNA causes a disturbance in the normal functioning of signaling pathways associated with the immune system, which, in turn, increases the risk for autoimmune complications. PDCD1 [67,68] is a programmed cell death gene that interrupts Runx1 binding due to an intronic enhanced SNP wherein such a circumstance has been found to occur in both T1D and SLE. In the same manner, a loss-of-Runx1 binding SNP was found to be associated with PSR between the NAT9 and SLC9A3R1 genes [69]. On the other hand, multiple NF-κB genes were deemed to be involved in the transcription processes of various autoimmune diseases. For instance, polymorphisms in the NFKB1 [70,71,72] gene have shown a direct link to T1D susceptibility in humans, while susceptibility in SLE is associated with the M196R polymorphism in TNFR2 [67,68,69,73]. Another transcription factor involved in T1D susceptibility is TCF7 [74], while PSR susceptibility includes IRF2 [75] and JUNB [76,77] found in the PSORS6 locus. IRF5 [78,79,80] and STAT4 [81,82] polymorphisms are present in both SSc and SLE, while the TBX21 [74,83] polymorphism is present in both SSc and T1D.

The enrichment results pertaining to the involvement of RNA polymerase II, chromatin, and ribosome suggest the relevance of the transcription process in autoimmunity. The occurrence of polymorphisms in the DNA found in chromatin may lead to the translation of dysfunctional proteins by rRNAs, especially transcription factors, that could affect the transcription of mRNAs and eventually cause immune system dysregulation. The binding affinity of transcription factors can be affected by SNPs found in the promoter region of a gene. Variations resulting from polymorphisms in these binding sites could interfere with the regular recruitment of transcription factors, causing deviations in gene expression levels. The regulatory network involved in gene expression may be compromised, which could result in either the upregulation or downregulation of the gene, which we has been found to be an important factor in autoimmune diseases. For instance, polymorphisms in the transcription of TLR genes have been found to cause malfunction in the signaling pathways crucial for the production of autoreactive T cells and B cells. A study by Assmann et al. [84,85,86,87,88,89] supported this thought when the authors confirmed the high correlation of TLR3 rs3775291 and rs13126816 polymorphisms with the risk of T1D development. Other studies verified the association of TLRs in the pathogenesis of SLE when they found that SNPs in TLR7, TLR8, and TLR9 increased SLE susceptibility in Asians [90,91], while SNPs in TLR3, TLR8, and TLR9 increased that in Danish subjects [92].

#### 4.2.2. Involvement of Post-Transcriptional Alternative Splicing in Autoimmunity Pathogenesis

Before proceeding to translation, newly synthesized mRNAs undergo post-transcriptional modifications such as capping, splicing, and polyadenylation. Alternative splicing increases the degree of diversity among species by producing multiple distinct mRNAs from a single gene. Since it is a highly regulated process, disruption in its mechanisms may negatively influence the maturation of mRNAs that could translate into dysfunctional proteins. Abnormal splicing events may cause mutations in the post-transcriptional processing of mRNA that could contribute to the dysregulation of autoimmune responses, such as the overactivation of T cells and B cells and the unregulated production and secretion of cytokines.

Using RNA sequencing and microarray analysis, Ergun et al. [93] discovered that about 60% of alternatively spliced isoforms are associated with lymphocyte genes. The involvement of alternative splicing in the complement protein C1 and complement receptors CR1 and CR2 has been found in bronchoalveolar macrophages and fibroblasts [94]. Isoforms of IL-2, IL4, and IL-6 have also been reported to have immunomodulation effects in the signal transduction of the immune response [95]. In connection to previous discussions, the occurrence of SNPs can result in genetic mutations that can lead to aberrant splicing events. The presence of SNPs may introduce new alternative binding sites or dislocate consensus splicing sites that could greatly influence the post-transcriptional modification of the mRNA [96]. Autoantigens, a group of chemoattractants responsible for recruiting immune cells to tissue damage sites, have been reported to be prone to alternative splicing [97]. These have been found to be significant in the initiation of autoantigen-specific autoimmune diseases, including the autoantigen islet cell Ag 512 in T1D [98]. Moreover, disruption in the splicing machinery may greatly influence SLE susceptibility, since it is mediated by the autoreactivity of T cells and B cells and their effect on the deposition of autoantibody–autoantigen complexes.

The enrichment results pertaining to mRNA splicing and the spliceosomal complex suggest that T1D, PSR, SSc, and SLE may have a common pathway relating to spliceosome-mediated post-transcriptional modification, which can be further studied for therapeutic strategy targeting. Table S3 lists the alternatively spliced isoforms of genes associated with the onset of the four autoimmune diseases.

#### 4.2.3. Role of Protein Phosphorylation in the Signal Transduction of Immune Dysregulation

Proteins typically generate post-translational modifications as a result of the physiological reaction to cellular stress, which can lead to the release of biological products, such as enzymes that facilitate the alteration of amino acid residues, and ROS [99]. The production of ROS in the body contributes to the alleviation of inflammation and regulation of tissue homeostasis. Nonetheless, excessive ROS release can cause these levels to rise to the point where the antioxidant ability of the body to remove excess ROS becomes dysfunctional [100]. Additionally, mutations induced by post-translational modifications can trigger the formation of neoepitopes, which the immune system deems foreign to the host and could lead to the disruption of immune tolerance, resulting in an amplified autoimmune response [101]. Post-translational modifications in “self” proteins can also affect T-cell and B-cell immunity such that alterations in APCs may influence immune system specificity [102].

Protein phosphorylation is a crucial mechanism in cell signaling pathways. This process refers to the transfer of an ATP-bound phosphate group to serine, threonine, or tyrosine amino acid residues in a substrate aided by the catalysis of a protein kinase. Kinases participate in this process by serving as enzymes in attaching the phosphate group from one protein to another to induce and maintain the transduction of signals in intracellular pathways. Aberrant protein phosphorylation events can participate in the increased risk of autoimmunity. Increased phosphorylation of STAT3, which is associated with manifestations in autoimmunity and immunodeficiency, has been correlated with multisystem autoimmune disease occurrence in humans. The subsequent dimerization of STAT–STAT protein interaction and nuclear translocation, as well as the increased phosphorylation of pY705 by Janus activating kinases (JAKs), activate STAT3 in response to chemokine signaling by IL-6, IL-10, IL-21, IL-23, and IFN-α [103]. High plasmatic concentrations of IL-6, IL-10, and IL-17 found in patients were believed to be associated with a novel mutation in STAT3, explaining its increased phosphorylation [104]. In another study, it was demonstrated that the inappropriate phosphorylation of IRF4 by ROCK2 downregulated production of IL-17 and IL-21 cytokines, which aggravated autoimmunity in mouse models. The aberrant activation of ROCK2 in CD4+ T cells negatively affected its ability to phosphorylate, which suggested its potential to ameliorate pathogenic mechanisms in initiating autoimmunity [105,106]. In the same manner, increased levels of CD44 phosphorylation have been linked to SLE susceptibility. This increased phosphorylation results in the increased expression of CD4, which, in turn, increases the ability of T cells in SLE to migrate to tissues and adhere to membranes [106].

Protein kinases phosphorylate proteins by converting extracellular signals into intracellular downstream readouts to initiate protein conformation and facilitate protein translocation. These are thought to be molecular precursors that activate self-reactive cells in the body that induce inflammation and compromise regulatory cells in autoimmune diseases. T cells, B cells, and other innate immune cells contain multifarious classes of immune recognition receptors that utilize phosphorylation to induce the initial activation of protein kinases. Receptor tyrosine kinases (RTKs), non-RTKs, receptor serine kinases (RSKs), and serine–threonine kinases (STKs) are the central kinases involved in the signal cascade of the immune response. JAK2 and TYK2 are members of the JAK family that are linked to multiple cytokine receptor signaling pathways in the pathogenesis of autoimmune disorders [107,108]. Moreover, these were also found to be present among the modules as far as this study is concerned, suggesting their common roles in the onset of T1D, PSR, SSc, and SLE.

#### 4.2.4. Regulatory Effects of T_H17_ in Autoimmune Diseases

**Figure 8:**
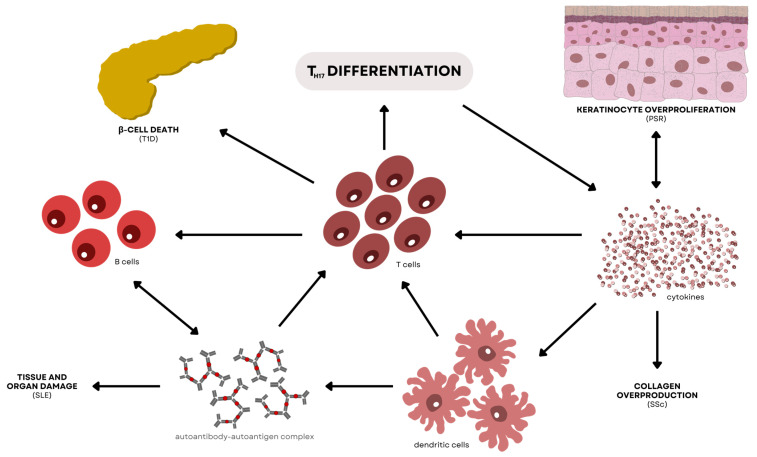
Interconnected pathways associated with T_H17_ differentiation involved in the pathogenesis of T1D, PSR, SSc, and SLE. In T1D, CD4+ T cells found in the pancreatic islets activate T_H17_ differentiation, which contributes to the increased production of IL-1β, IL-6, and TNF-α. These cytokines allow the easy penetration of CD4+ and CD8+ T cells in the pancreas, allowing the direct killing of β cells. In PSR, the secretion of IL-17A and IL-22 causes the migration of more T_H17_ cells in the inflammation site, which results in epidermal acanthosis. The activation of mDCs, which is one of the triggers in PSR, initiates T_H17_ differentiation to continue producing the cytokines responsible for maintaining the cyclic overgrowth of keratinocytes. In SSc, T_H17_ cells promote fibrosis of the skin and lungs by activating the production and stimulation of collagen. SSc incidence also shows a correlation with IL-22, which is a pro-inflammatory cytokine found in the skin. Lastly, activated CD4+ T cells may play a role in the elevated levels of IL-17 in SLE patients. Overexpression of the IL-17 gene was also found to be associated with the manifestation of the disease.

Most of the modules from the KEGG pathway results showed association with T_H17_ differentiation, which may imply the significant participation of T_H17_ cells in the simultaneous onset of T1D, PSR, SSc, and SLE. Apart from the classic T_H1_ and T_H2_ subsets, T_H17_ cells are a more recently discovered T-helper subset that aid in the specificity of the immune system. These immune cells preferentially contribute to the production and secretion of IL-17A, IL-17F, IL-21, and IL-22 [109]. The pathophysiology of numerous autoimmune disorders is influenced by T_H17_ cells and their effector cytokines, which also mediate host defensive mechanisms against a variety of infections, particularly extracellular bacterial infections. It is important to emphasize that the IL-17R and IL-22R receptors are widely expressed on a variety of epithelial tissues in the human body [110], which reasons out why T_H17_ cell effector cytokines are essential for tissue immunity and for arbitrating critical immune system–tissue interaction. Figure 8 shows the interconnected pathways of the four diseases central in T_H17_ differentiation.

In non-obese diabetic (NOD) mice, it has been found that there is a correlation between insulitis and IL-17 and IL-17F expression in the islets of Langerhans in the pancreas. Although the exact pathogenesis of T1D is, as yet, unclear, studies have proven that the disease involves the unregulated activation of several immune cells, including the participation of B cells, DCs, macrophages, and CD4+ and CD8+ T cells [111,112,113]. The stimulation of β-cell autoantigens activates the differentiation and proliferation of T_H17_ cells by autoreactive CD4+ T cells in the islets. This then causes the production and secretion of IL-17, which enhances the release of pro-inflammatory cytokines IL-1β, IL-6, and TNF-α. These cytokines have a direct effect on the immunogenicity of the pancreatic islets and the survival and apoptosis of β cells. These cytokines also enable CD4+ and CD8+ T cells to infiltrate β cells and aggravate their killing. Studies on the disease have concluded that the primary function of T_H17_ cells is in the induction of inflammatory processes in T1D, resulting in the death of pancreatic β cells [111,112]. Meanwhile, the action of IL-17 alone or its synergy with IFN-γ or IL-1β induces heightened expression of nitric oxide synthase-2A, cyclooxygenase-2, and superoxide dismutase-2, which are said to participate in the inflammation of the pancreatic islets [114]. This concludes the main function of IL-17 in T1D, that is, to aggravate such inflammatory effects on β cells, leading to their destruction.

The explicit role of TNF in the pathogenesis of psoriasis has been established by its significance as a blocker treatment for the disease. TNF is produced by various T lymphocytes, including T_H1_ and T_H17_ cells, and keratinocytes, which have both TNF-α and IL-17 receptors that can induce inflammatory responses upon activation. A common route between TNF and IL-17 signaling is the stimulation of NF-κB. Recent studies have shown that TNF-α may activate the T_H17_ response indirectly by stimulating mDCs [111,115]. Also, the synergistic interaction of TNF-α and IL-17 has been recorded to activate certain genes found in fibroblast and osteoblast cells. Furthermore, a study by Zaba et al. reported the potential of psoriatic dermal DCs to induce T-cell proliferation and polarization, aiding in the differentiation of T_H17_ and T_H1_ cells [116]. The release of IL-17A and IL-22 triggers the expression of CCL20, which is a keratinocyte-expressing ligand, which causes the chemotaxis of more T_H17_ cells to the inflammation site and drives epidermal acanthosis, suggesting early manifestations of PSR. In the presence of TGF-β and IL-6, naïve T cells undergo T_H17_ development due to cytokines IL-23, IL-12, and TNF-α being released by activated mDCs in response to physical trauma [117]. Moreover, ROR-γt and ROR-α expression, as well as STAT3 activation, are necessary for T_H17_ differentiation. Pro-inflammatory cytokines, including IL-17A, IL-17F, and IL-22, then cause the abnormal differentiation and proliferation of keratinocytes, leading to the production and secretion of chemokines, angiogenic factors, and antimicrobial peptides [118]. These mediators are able to draw immune cells to the skin lesions, activate them, and create a positive feedback loop that aggravates the core response of PSR.

Several studies have found that there is an increased level of T_H17_ cells and their products in the blood and skin among SSc patients compared to healthy controls [119,120,121,122]. Some findings, on the other hand, showed evidence of the association of T_H17_ cells with certain manifestations of SSc, such as collagen overproduction and lung impairment [123,124]. Using murine SSc models, it was reported that IL-17A/T_H17_ cells can promote fibrosis of the skin and lungs by stimulating the progression and secretion of type 1 collagen [120,125]. However, some researchers found that IL-17A/T_H17_ cells downregulated type 1 collagen production upon the differentiation of fibroblasts into myofibroblasts, concluding that said mechanism may not be driven by fibrotic activity but by autoimmunity [126,127,128]. Even though this result is indicative of anti-fibrosis events, such a pathway is deemed contrary to the pro-fibrotic activity of other immune cells that still support the fibrotic manifestations in SSc. In addition to the IL-17 family, other T_H17_-derived cytokine members also have clear correlations with SSc, one of which is the production and secretion of IL-22, a crucial pro-inflammatory cytokine in the skin because of its increased ability to destroy bacteria and produce chemokines, such as IL-8 and MCP-1, as well as cytokines TNF, IL-1, and IL-12 [129]. However, a different study discovered that while IL-21 is high, serum IL-23 and IL-17 are also reduced [120,126,130,131,132]. Either way, it is undeniable that these cytokines produced from T_H17_ play a pivotal role in SSc expression.

Increased plasma or serum levels of IL-17 were also seen in patients with SLE, even those who at the early stages of the disease [133,134]. Studies have shown that the circulation of this cytokine in the body could be associated with the expression of SLE [133,134,135]. Levels of IL-17 were even higher in SLE patients with nephritis relative to those without. IL-17 can be produced by several kinds of immune cells linked to SLE, including CD3+, CD4−, and CD8− T cells; CD4+ and CD8+ T cell; γδ-T cells; and natural killer cells [134]. Accordingly, it is speculated that the increased levels of IL-17 in the blood could be due to the enhanced production of the cytokine by these immune cells [135]. However, it was found that the expression of T_H17_ cells is not correlated with the expression of T_H1_ cells in SLE-positive blood. In other words, it is still uncertain whether the frequency of IL-17-producing T_H17_ cells has an influence on the frequency of IFN-γ-producing T_H1_ cells. Nonetheless, this was further tested by analyzing the ratio of IL-17-producing CD4+ T cells to T_H1_ cells among the patients with devoid changes in the T_H17_/T_H1_ ratio. Results showed that the T_H17_/T_H1_ ratio was still higher in SLE patients compared to healthy controls, suggesting that unregulated activation in CD4+ T cells could play a role in the stimulated increase in frequency of T_H17_ differentiation in SLE [136,137]. As mentioned in previous discussions, IL-17 is an essential component in the release of inflammatory cytokines in the immune response. Overexpression of the IL17 gene has been observed in the urine sediments of SLE patients, further confirming its participation in SLE pathogenesis, especially in tissue damage [138]. Blocking of the IL-17 signal has also been linked with reduced development and proliferation of B cells, indicating the impact of this disease on the production of antibodies.

### 4.3. Drug Repositioning for Immune Dysregulation Treatment

The top 50 hub genes from each module were obtained through cytoHubba using the Cytoscape software. The overlapping hub genes among the modules were determined, grouped either as upregulated or downregulated, and subjected to DRA. Clomiphene, an estrogen receptor agonist, was the top drug in the upregulated group, while prilocaine, a local anesthetic, was the top drug in the downregulated group.

#### 4.3.1. Drug Repurposing Analysis of Upregulated Hub Genes

Clomiphene, estrone, and norethindrone are drugs associated with the main female sex hormones, i.e., estrogen and progesterone. Scientific evidence has shown the prevalence of both estrogen and progesterone receptors in various immune response mechanisms. CD4+ and CD8+ T cells, B cells, and NK cells found in human peripheral blood contain intracellular estrogen receptors, specifically ERα46, which is its commonly expressed isoform [139]. Estrogen also controls B cell maturation by inhibiting or downregulating the differentiation of pro-B cells to pre-B cells, which influences their rate of survival and activity in stimulating autoreactive B cells. In vitro studies have confirmed the anti-psoriatic functions of estrogen in regulating the expression of PSR [140,141]. Recall that the overproliferation of keratinocytes in PSR is activated by chemokines and AMPs. For instance, 17β-estradiol (E2) downregulates the cyclic activation of keratinocyte production by binding to estrogen receptors in immune cells, which results in a decrease in MAPK, STAT3, PI3K, and NF-κB activity, regulating the stimulation of these chemokines and AMPs [142].

With clomiphene as the top drug obtained from the upregulated hub genes, this result suggests that the expression of estrogen receptors in T1D, PSR, SSc, and SLE may be a triggering effect, which reasons out the need for its inhibition among the four diseases. In T1D, it has been shown that estradiol can contribute to an increase in insulin content in β cells [143]. Additionally, a reduction in circulating estrogen can predispose to a higher risk of T1D incidence [144]. It is assumed that the presence of estrogen receptors can initiate the digestion of estrogen, which could significantly decrease its number. Thus, inhibition of these receptors may mitigate the depletion of the hormone so that it could perhaps counteract the inability of the pancreas to secrete insulin. Instead of binding to its receptors, estrogen may participate in pathways that aid in the preservation or synthesis of insulin to conciliate T1D ramifications. Moreover, the interaction of estrogen with ERβ could be the reason for the increased mannan-induced skin inflammation in PSR. Some effects of such a response include thickness of the epidermis, expression of *cebpb*, infiltration of DCs and γδ-T cells, and stimulation of pro-inflammatory cytokines and chemokines [145]. These could also serve as significant evidence of the possibility of inhibiting estrogen receptors for the treatment of PSR. However, in SSc, a study discovered that estrogen utilizes ERα to alleviate dermal fibrosis by the inhibition of TGF-β [146]. With that, clomiphene may be repurposed to act only on ERβ to increase the efficiency of estrogen to bind with ERα and become more effective in treating SSc-mediated fibrosis. Lastly, another study reported that the autoantigenicity of ER is greatly enhanced among SLE patients, explaining its association with mitochondrial dysfunction by the formation of estrogen–ER complexes [147]. To address this, estrogen receptors could be inhibited to prevent the synthesis of these complexes to avoid immune attacks induced by SLE.

#### 4.3.2. Drug Repurposing of Downregulated Hub Genes

Escitalopram and piracetam are drugs whose pharmacodynamics involve the regulation of neurotransmitter activity, while the mechanistic action of the top drug, prilocaine, blocks the propagation of action potential by acting on the neuronal cell membrane. Serotonin has long been known to have immunomodulatory effects in the body. The release of serotonin by mast cells activates 5-HT_2_ receptors in T cells, which causes delayed hypersensitivity, and its stimulatory effect on 5-HT_7_ receptors enhances the development of naïve T cells [148]. Moreover, serotonin influences adaptive immunity by aiding B-cell proliferation by activating 5-HT_1A_ receptors [149]. In some cases, the inhibition of serotonin reuptake has been found to increase circulating B cells. However, the direct implication of serotonin in autoimmunity has yet to be elucidated, although its molecular machinery in alleviating or aggravating autoimmune effects in homeostasis has already been given attention in the scientific community.

The pharmacodynamics of prilocaine involve the binding of the drug to sodium channels, which prevents the influx of sodium ions into the cell, thereby inhibiting the intracellular propagation of action potential. The metabolism of glucose acts on various membrane proteins, such as ion channels, which initiates a cascade of electrical activity that has a significant impact on the release of insulin [150]. A common clinical symptom in diabetes is diabetic neuropathy, which is characterized by the damage to peripheral nerves due to hyperglycemia [151]. Since prilocaine is an inhibitory drug of action potential propagation, which is contrary to the supposed increased electrical signaling to induce greater insulin release in treating hyperglycemia, it may be repositioned to attain stimulatory effects in activating the action potential but without compromising its specificity to sodium ion channels. Furthermore, it was discussed that common pathologic manifestations of PSR, SSc, and SLE are correlated with skin inflammation. Several studies have reported that blocking sodium channels may improve the skin’s barrier functions to inflammatory responses [152,153,154,155,156,157]. These autoimmune diseases may involve pathways that could lead to sodium dysregulation and negatively affect the sodium homeostasis of the body. As mentioned above, the participation of TNF-α in skin inflammation is very prevalent in such a way that most autoimmune diseases involving skin conditions are associated with it. Local anesthetics directly influence TNF-α production, predisposing it as candidate for the use suppressive drugs in downregulating the overactive inflammatory response in autoimmune diseases. This suggests that prilocaine, similar to other local anesthetics such as lidocaine and procaine, may be utilized for the treatment of PSR, SSc, and SLE. However, modifications may still be required to change the drug’s mechanism of action for the treatment of T1D.

## 5. Conclusions

The complex nature of autoimmunity makes it a challenging problem for the scientific community to address. The interplay between genetic and environmental factors in the manifestation of autoimmune diseases continues to be an unsolved link in designing the most effective and efficient drugs and therapies. To determine the molecular and cellular mechanisms responsible for the interconnected pathogenesis of autoimmune disorders, we looked into the expression patterns of these diseases and analyzed the pathways that may elucidate their correlation. Using WGCNA, we identified thirteen highly preserved modules of co-expressed genes based on the microarray datasets of T1D, PSR, SSc, and SLE. The functional annotation of the modules revealed that the clusters of co-expressed genes have associations with the transcription, post-transcription, and post-translational processes occurring among the four diseases. The KEGG pathway result also suggested the role of T_H17_ differentiation in the possible interplay of the diseases. On the other hand, clomiphene and prilocaine were the top candidate drugs in regulating overexpressed and under-expressed hub genes, respectively. These findings shed light on the possible routes that could be targeted for the engineering of treatments to medicate the possible and simultaneous onset of T1D, PSR, SSc, and SLE.

Since the datasets obtained from GEO and the literature supporting the relationships of autoimmune diseases were limited, only T1D, PSR, SSc, and SLE were included in the analysis. RNA-Seq data, which also hold promising information in transcriptomics analyses, may also be utilized instead of DNA microarray data. Future researchers may look into other autoimmune disorders and elucidate their interconnectedness using WGCNA. They may also investigate individual genes obtained from the modules, which is contrary to the objectives of this study, which focused on the systemic relationship among the augmented genes. Drug repurposing may be further evaluated by using receptor–ligand bioinformatics to procure a deeper understanding of the mechanistic action of the drugs for a more reliable treatment process.

## Figures and Tables

**Table 1:** The parameters used in identifying the GEO datasets for the study.

	T1D (GSE35725)	PSR (GSE55201)	SSc (GSE65336)	SLE (GSE61635)
**Organism**	*H. sapiens*			
**Sample Type**	Blood			
**Experiment Type**	Expression profiling by array			
**Platform**	Affymetrix Human Genome U133 Plus 2.0 Array (GPL570)			
**No. of Positive Samples**	44	44	29	64
**No. of Negative Controls**	44	30	29	30

**Table 2:** The top 5 drug candidates obtained from DRE for regulating abnormal expression of hub genes found among the diseases.

Expression	Hub Genes	Drug Name	Mechanism	Tau	FDR
Upregulated	*CD8A*, *CCL5*, *TP53*, *MED1*, *CD4*, *SYK*, *BCL2*, *PRKCA*, *GNB1*, *HSP90AB1*, *PIK3R1*, *SMAD3*, *TOP2A*, *FYN*, *CDK2*, *MRPL3*, *RPL35*, *RPS5*, *RPS24*, *CD40*, *IMP3*	Clomiphene	Estrogen receptor antagonist	−97.3	0.00003
		Estrone	Estrogen receptor agonist, estrogenic hormone	−96.7	0.00635
		Trimethobenzamide	Histamine receptor antagonist	−96.6	0.00292
		Norethindrone	Progesterone receptor agonist	−96.5	0.00353
		Cladribine	Adenosine deaminase inhibitor, ribonucleotide reductase inhibitor	−96.3	0.00415
Downregulated	*CD44*, *BRCA1*, *TLR4*, *ITGAM*, *STAT1*, *MYC*, *JUN*, *CASP3*, *CCNA2*, *FOS*, *MAPK1*, *CXCR4*, *CCL2*, *MAPK14*, *TLR2*, *CXCL8*, *TGFB1*, *IL1B*, *ICAM1*, *MAPK3*, *APOE*, *MMP9*, *PTPRC*, *JAK2*, *GSK3B*, *CTNNB1*, *EZH2*, *DDX58*, *PTEN*	Prilocaine	Local anesthetic	−99.5	0.00544
		Montelukast	Leukotriene receptor antagonist	−99.2	0.00662
		Escitalopram	Selective serotonin reuptake inhibitor	−99.1	0.00566
		Piracetam	Acetylcholine receptor agonist	−99.0	0.00003
		Oxymetholone	Androgen receptor agonist	−98.2	0.00398

**Table 3:** Summary of the mechanistic effect of immune dysregulation on the pathogenesis of T1D, PSR, SSc, and SLE.

Disease	Effect of Immune Dysregulation	Ref.
T1D	Unrestricted immune cells initiate a series of immunologic effects that target β cells, resulting in the inefficiency of the pancreas in producing insulin in response to elevated glucose levels in the blood.	[51,52,53]
PSR	The immune system enters an unregulated, continuous cycle where the overstimulation of keratinocytes produces chemokines and AMPs that, in turn, activate the immune cells to induce further proliferation of skin cells.	[54,55,56]
SSc	Vascular triggers result in the overproduction of ECM, which usually accumulates in connective tissues, due to the uncontrolled release of cytokines by immune cells.	[57,58,59]
SLE	The abnormal function of immune cells causes the formation of autoantibody–autoantigen complexes that form an immunologic cycle, which creates an inflammatory response where the immune system starts to attack the body’s tissues and organs.	[60,61,62]

## Data Availability

The data and results presented in this study are available upon request from the first and corresponding authors.

## Supplementary Materials

The following supporting information can be downloaded at: https://www.mdpi.com/article/10.3390/genes15040393/s1, Figure S1: General network properties of the datasets for comparability testing prior to WGCNA: (**a**) T1D vs. PSR; (**b**) T1D vs. SSc; (**c**) T1D vs. SLE; (**d**) PSR vs. SSc; (**e**) PSR vs. SLE; (**f**) SSc vs. SLE. All the plots exhibited positive correlation which suggests the presence of co-expression genes among the datasets; Figure S2: An integrated visual of the dendrogram from gene clustering through TOM dissimilarity and the module split sensitivity networks through the dynamic tree-cutting algorithm of the reference dataset. The deep split parameter used in this study is enclosed in a red box; Figure S3: Protein-protein interaction networks of the overlapping hub genes in the 13 modules: (**a**) brown module; (**b**) cyan module; (**c**) dark turquoise module; (**d**) green yellow module; (**e**) grey60 module; (**f**) light green module; (**g**) light yellow module; (**h**) magenta module; (**i**) midnight blue module; (**j**) red module; (**k**) salmon module; (**l**) tan module; (**m**) turquoise module; Table S1: The top 5 terms in the BP, CC, MF, and KEGG annotations from each module obtained from the functional annotation clustering; Table S2: List of overlapping hub genes from each module obtained from the three algorithms; Table S3: List of alternatively spliced isoforms found to participate in T1D, PSR, SSc, and SLE susceptibility. References [158,159,160,161,162,163,164,165,166,167,168,169,170,171,172,173,174,175,176,177,178,179,180,181,182,183,184,185,186,187,188,189,190,191] are cited in the supplementary materials.
