# Supplementary material for: Theoretical Studies of DNA Microarray Present Potential Molecular and Cellular Interconnectivity of Signaling Pathways in Immune System Dysregulation
Source: Genes (Basel). 2024 Mar 22;15(4):393. doi: 10.3390/genes15040393 (PMC11049615; doi:10.3390/genes15040393)
Supplement: Supplementary file 1 [file genes-15-00393-s001.zip › genes-2897456-supplementary.pdf]

SUPPLEMENTARY MATERIAL

Theoretical Studies of DNA Microarray Present Potential Molecular and Cellular Interconnectivity of Signaling Pathways in Immune System Dysregulation

Jon Patrick T. Garcia and Lemmuel L. Tayo

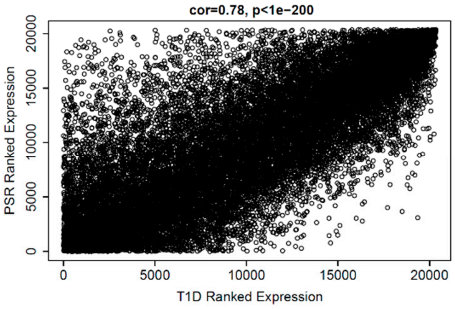

(**a**)

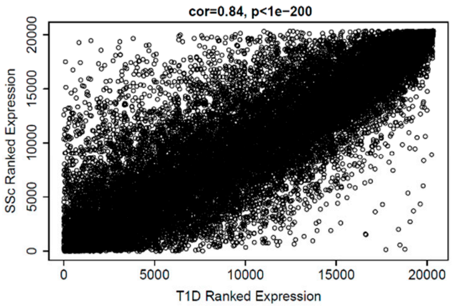

(**b**)

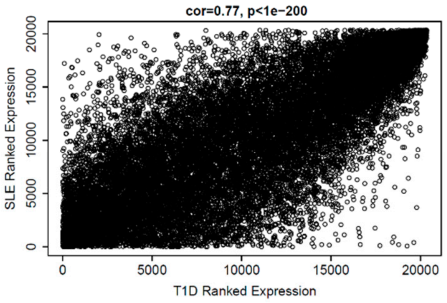

(**c**)

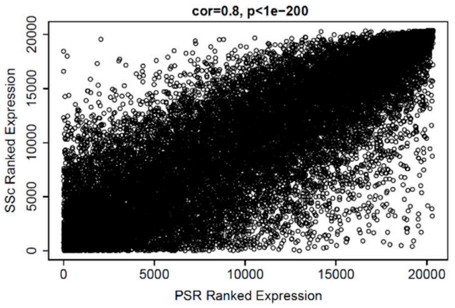

(**d**)

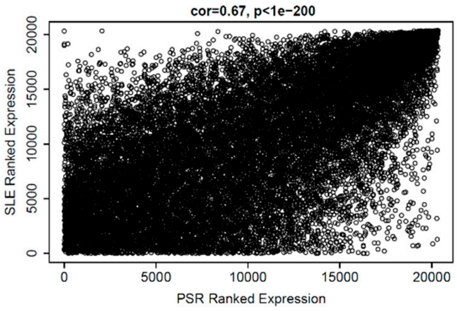

(**e**)

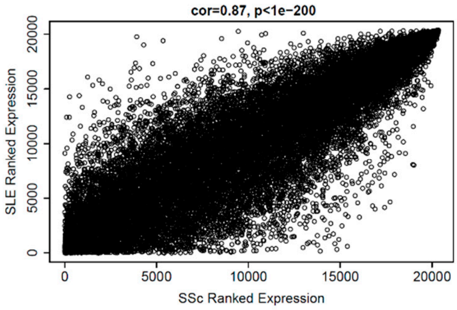

(**f**)

Figure S1. General network properties of the datasets for comparability testing prior to WGCNA: (**a**) T1D vs PSR; (**b**) T1D vs SSc; (**c**) T1D vs SLE; (**d**) PSR vs SSc; (**e**) PSR vs SLE; (**f**) SSc vs SLE. All the plots exhibited positive correlation which suggests the presence of co-expression genes among the datasets.

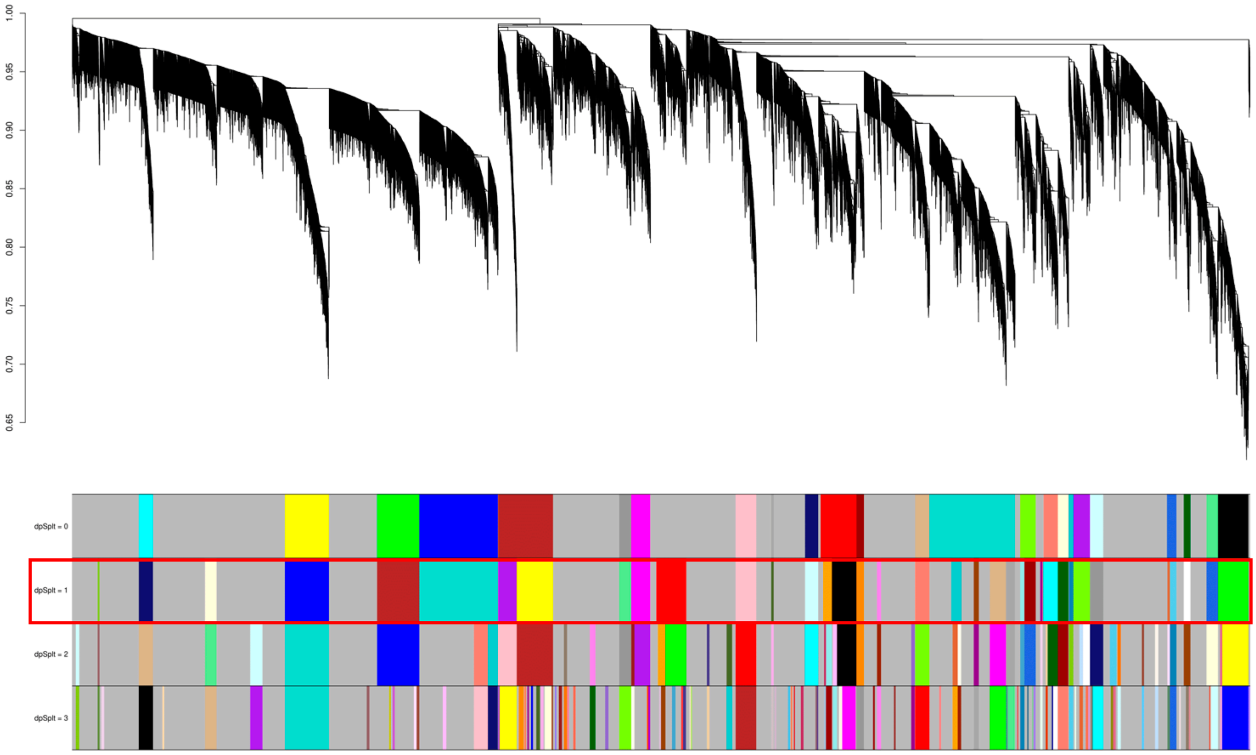

Figure S2. An integrated visual of the dendrogram from gene clustering through TOM dissimilarity and the module split sensitivity networks through the dynamic tree-cutting algorithm of the reference dataset. The deep split parameter used in this study is enclosed in a red box.

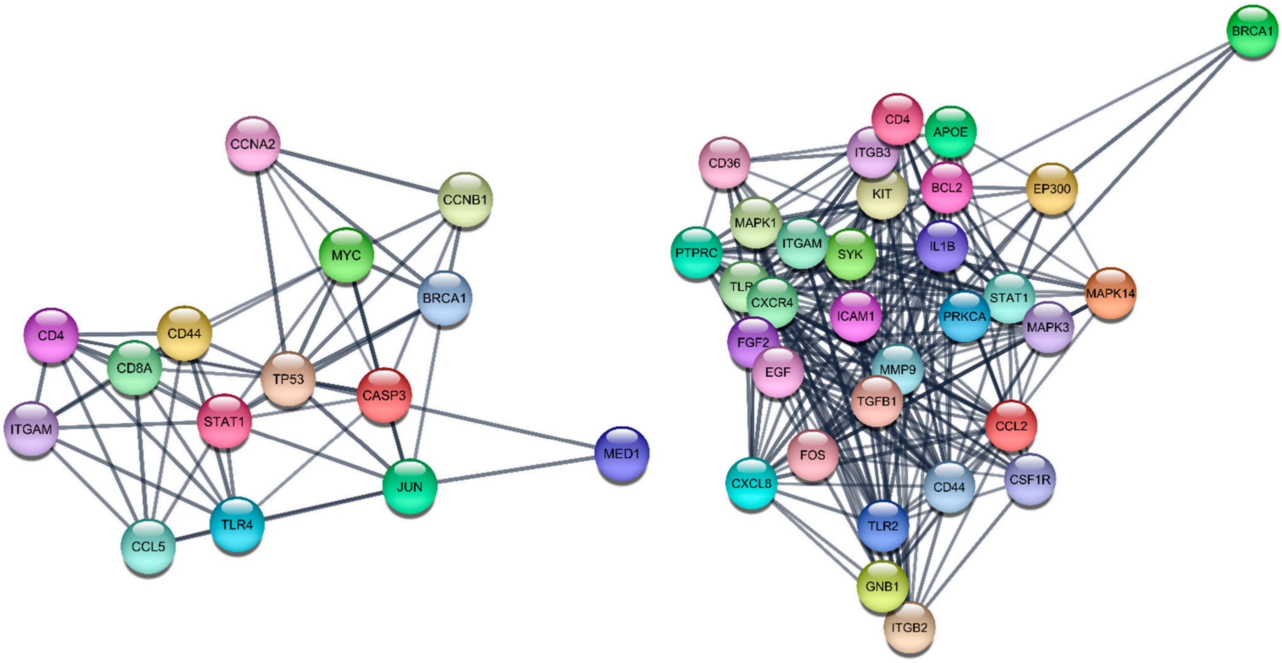

(**a**) (**b**)

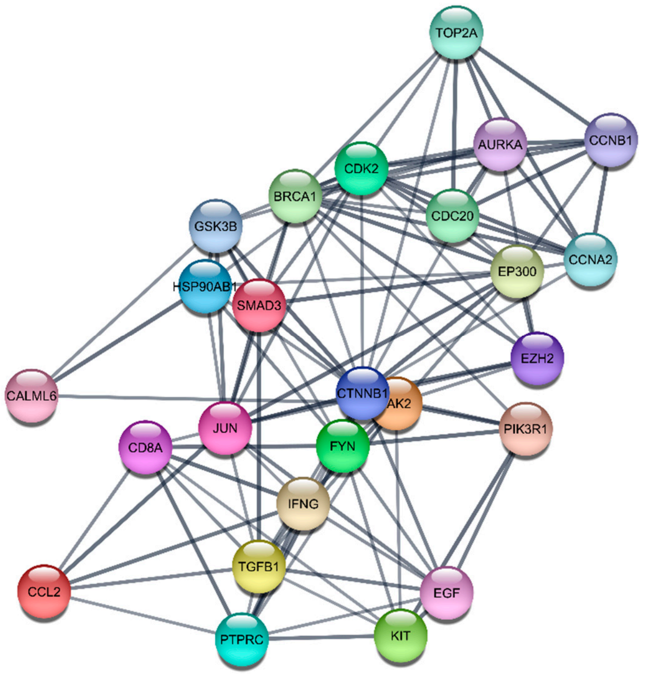
TOP2A
AURKA
CCNB1
CDK2
BRCA1
CDC20
GSK3B
CCNA2
EP300
HSP90AB1
SMAD3
EZH2
CALML6
CTNNB1
AK2
PIK3R1
CD8A
JUN
FYN
IFNG
TGFB1
CCL2
EGF
PTPRC
KIT

(c)

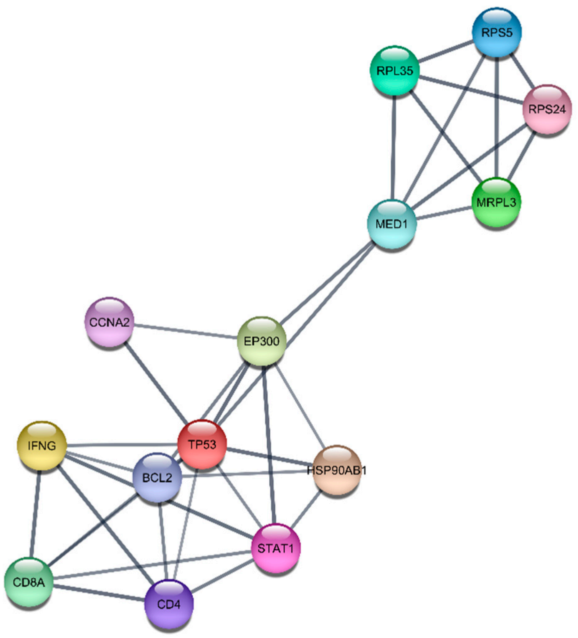
RPS5
RPL35
RPS24
MRPL3
MED1
CCNA2
EP300
IFNG
TP53
BCL2
HSP90AB1
STAT1
CD8A
CD4

(d)

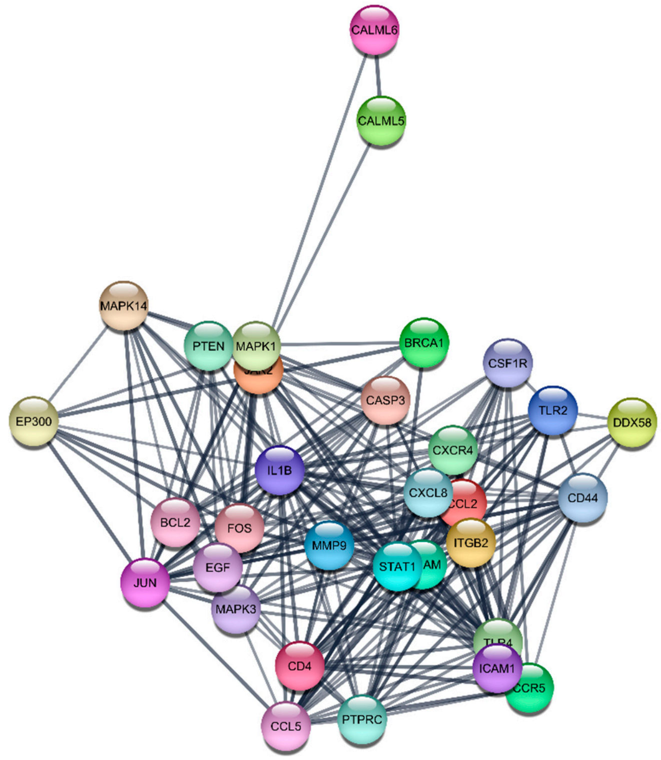
CALML6
CALML5
MAPK14
PTEN
MAPK1
BRCA1
CSF1R
CASP3
TLR2
DDX58
EP300
CXCR4
IL1B
CXCL8
CL2
CD44
BCL2
FOS
MMP9
ITGB2
JUN
EGF
STAT1
AM
MAPK3
TLR4
CD4
ICAM1
CCR5
CCL5
PTPRC

(e)

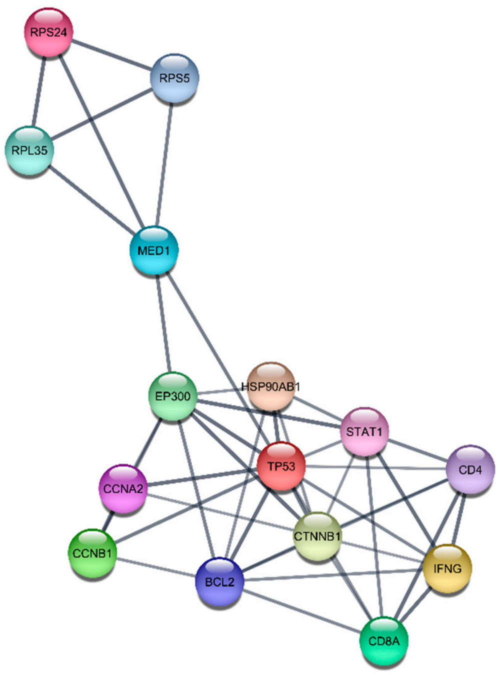
RPS24
RPS5
RPL35
MED1
EP300
HSP90AB1
STAT1
TP53
CD4
CCNA2
CTNNB1
CCNB1
BCL2
IFNG
CD8A

(f)

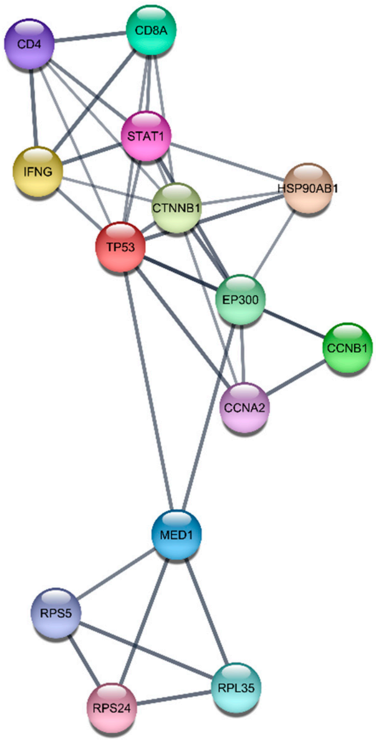
CD4
CD8A
STAT1
IFNG
HSP90AB1
CTNNB1
TP53
EP300
CCNB1
CCNA2
MED1
RPS5
RPL35
RPS24

(**g**)

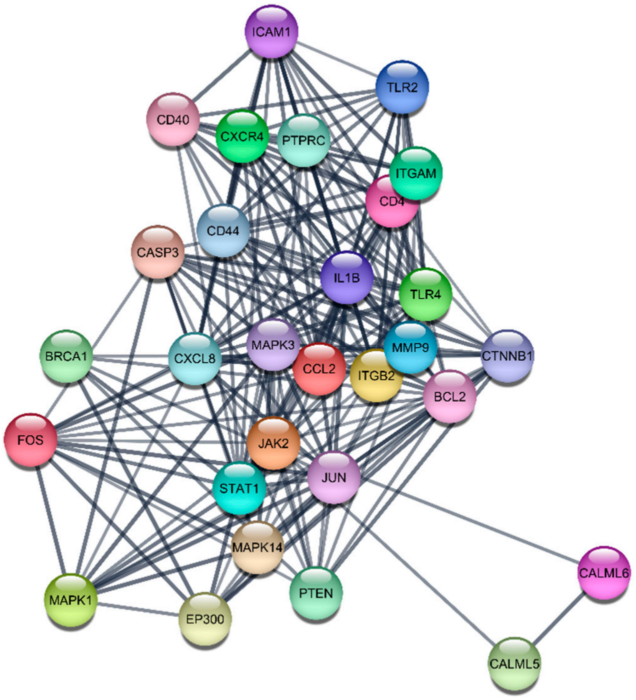
ICAM1
TLR2
CD40
CXCR4
PTPRC
ITGAM
CD4
CD44
CASP3
IL1B
TLR4
BRCA1
CXCL8
MAPK3
CCL2
ITGB2
MMP9
CTNNB1
BCL2
FOS
JAK2
STAT1
JUN
MAPK14
CALML6
MAPK1
EP300
PTEN
CALML5

(**h**)

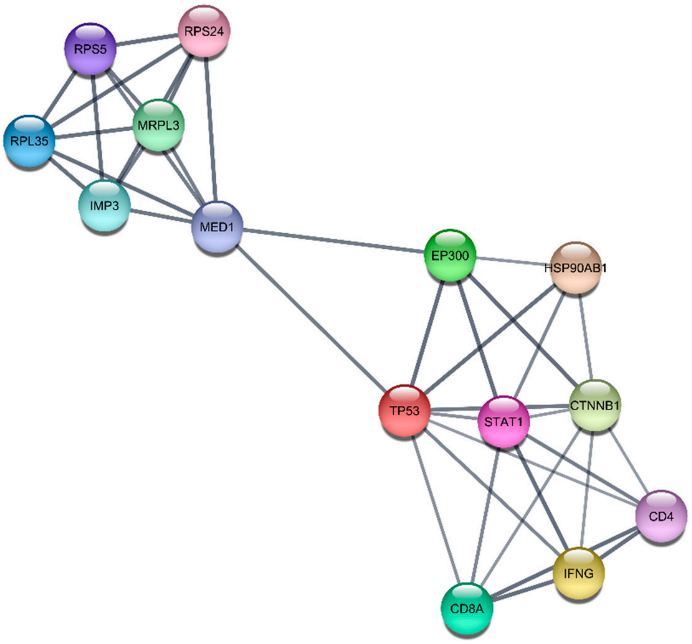
RPS5
RPS24
MRPL3
RPL35
IMP3
MED1
EP300
HSP90AB1
TP53
STAT1
CTNNB1
CD4
IFNG
CD8A

(**i**)

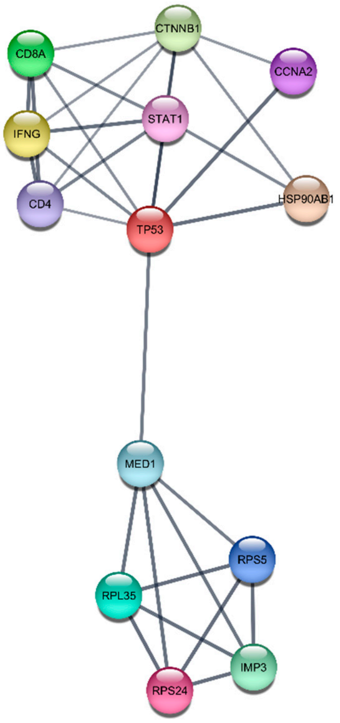
CTNNB1
CD8A
CCNA2
STAT1
IFNG
HSP90AB1
CD4
TP53
MED1
RPS5
RPL35
IMP3
RPS24

(**j**)

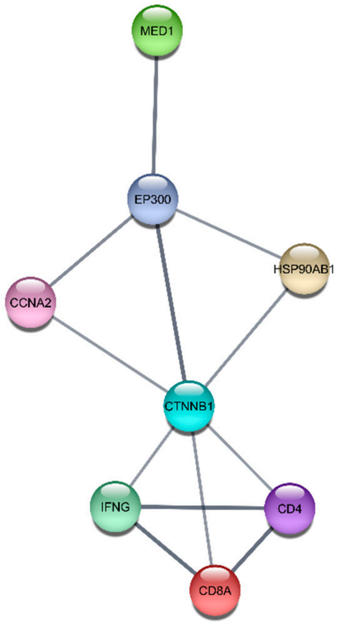

(**k**)

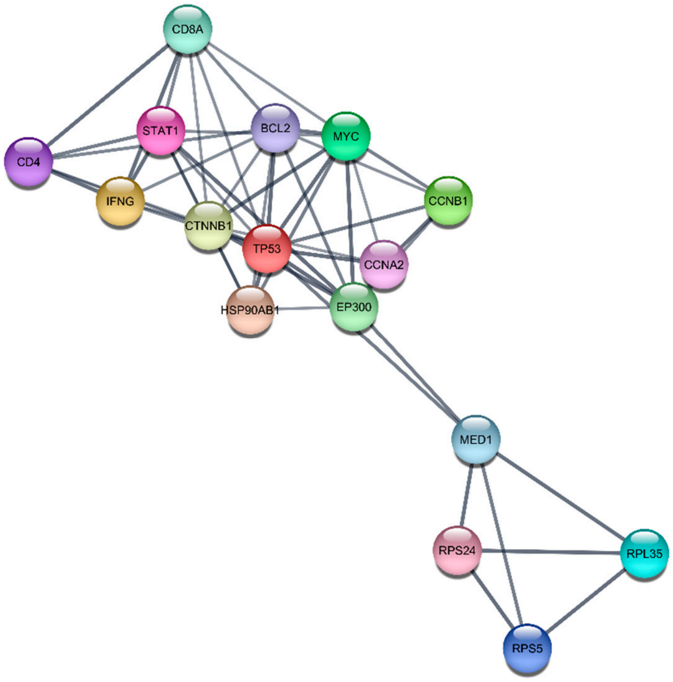

(**l**)

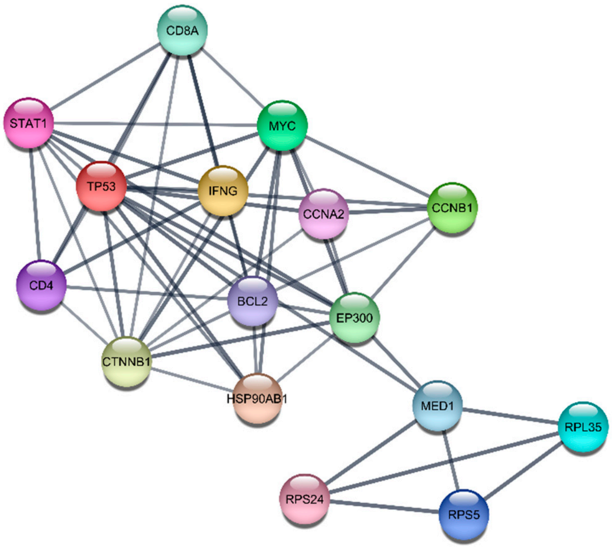

(**m**)

Figure S3. Protein-protein interaction networks of the overlapping hub genes in the 13 modules: (**a**) brown module; (**b**) cyan module; (**c**) dark turquoise module; (**d**) green yellow module; (**e**) grey60 module; (**f**) light green module; (**g**) light yellow module; (**h**) magenta module; (**i**) midnight blue module; (**j**) red module; (**k**) salmon module; (**l**) tan module; (**m**) turquoise module.

Table S1. The top 5 terms in the BP, CC, MF, and KEGG annotations from each module obtained from the functional annotation clustering.

Module	Annotation	Term	Count	Adj. *p*-value
brown	BP	GO:0006468 protein phosphorylation	103	1.5E-7
		GO:0070098 chemokine-mediated signaling pathway	26	4.0E-6
		GO:0030593 neutrophil chemotaxis	25	2.9E-4
		GO:0002548 monocyte chemotaxis	15	2.4E-3
		GO:0006177 GMP biosynthetic process	8	2.8E-5
	CC	GO:0000785 chromatin	196	6.0E-5
		GO:0005777 peroxisome	25	1.4E-2
		GO:0005778 peroxisomal membrane	19	3.3E-3
		GO:0030658 transport vesicle membrane	14	3.5E-3
		GO:0008305 integrin complex	9	2.8E-2
	MF	GO:0004712 protein serine/threonine/tyrosine kinase activity	99	8.9E-6
		GO:0004672 protein kinase activity	81	3.4E-4
		GO:0004674 protein serine/threonine kinase activity	80	2.2E-3
		GO:0030527 structural constituent of chromatin	28	4.6E-4
		GO:0008234 cysteine-type peptidase activity	14	1.9E-3
	KEGG	hsa05162 measles	42	1.2E-3
		hsa04658 Th1 and Th2 cell differentiation	29	4.7E-3
		hsa04659 Th17 cell differentiation	29	4.2E-2
		hsa05321 inflammatory bowel disease	20	2.7E-2
		hsa04940 type 1 diabetes mellitus	14	4.8E-2
cyan	BP	GO:0045944 positive regulation of transcription by RNA polymerase II	258	3.0E-4
		GO:0006468 protein phosphorylation	96	1.5E-6
		GO:0030593 neutrophil chemotaxis	32	3.2E-8
		GO:0098869 cellular oxidant detoxification	28	7.9E-7
		GO:0033674 positive regulation of kinase activity	21	9.0E-4
	CC	GO:0000785 chromatin	212	4.1E-9
		GO:0000786 nucleosome	30	7.6E-3
		GO:0031838 haptoglobin-hemoglobin complex	8	5.2E-4
		GO:0005833 hemoglobin complex	7	5.6E-3
		GO:1990779 glycoprotein Ib-IX-V complex	5	1.3E-3
	MF	GO:0004714 transmembrane receptor protein tyrosine kinase activity	83	6.2E-3
		GO:0004712 protein serine/threonine/tyrosine kinase activity	100	8.9E-7
		GO:0004674 protein serine/threonine kinase activity	88	1.1E-5
		GO:0004672 protein kinase activity	81	9.7E-5
		GO:0004713 protein tyrosine kinase activity	27	6.8E-3

	KEGG	hsa04270 vascular smooth muscle contraction	41	2.4E-4
		hsa04921 oxytocin signaling pathway	39	1.3E-2
		hsa04728 dopaminergic synapse	35	9.5E-3
		hsa04260 cardiac muscle contraction	17	4.4E-2
		hsa05216 thyroid cancer	10	1.8E-2
dark turquoise	BP	GO:0006468 protein phosphorylation	107	4.5E-3
		GO:0007169 transmembrane receptor protein kinase signaling pathway	35	1.6E-4
		GO:0006935 chemotaxis	29	3.1E-3
		GO:0000079 regulation of cyclin-dependent protein serine/threonine kinase activity	13	4.6E-2
		GO:0035825 reciprocal DNA recombination	5	3.3E-3
	CC	GO:0000307 cyclin-dependent protein kinase holoenzyme complex	14	3.6E-3
		GO:0000794 condensed nuclear chromosome	8	2.7E-3
		GO:00005861 troponin complex	6	2.9E-3
		GO:0070533 BRCA1-C complex	5	3.4E-3
		GO:1990779 glycoprotein Ib-IX-V complex	4	1.8E-2
	MF	GO:0004712 protein serine/threonine/tyrosine kinase activity	103	8.5E-8
		GO:0004713 protein tyrosine kinase activity	27	6.1E-3
		GO:0004714 transmembrane receptor protein tyrosine kinase activity	16	5.2E-3
		GO:0016493 C-C chemokine receptor activity	11	5.0E-4
		GO:0004950 chemokine receptor activity	10	4.5E-4
	KEGG	hsa04931 insulin resistance	23	1.1E-2
		hsa04666 Fc gamma R-mediated phagocytosis	20	1.7E-2
		hsa05142 Chagas disease	18	4.3E-2
		hsa05332 graft-versus-host disease	14	9.4E-3
		hsa04940 type 1 diabetes mellitus	10	2.2E-2
green yellow	BP	GO:0006468 protein phosphorylation	93	8.0E-5
		GO:0007186 G-protein coupled receptor signaling pathway	51	1.0E-2
		GO:0000398 mRNA splicing, via spliceosome	43	4.9E-3
		GO:0006413 translation initiation	19	3.7E-4
		GO:0006913 nucleocytoplasmic transport	19	2.2E-4
	CC	GO:0005681 spliceosomal complex	37	5.1E-5
		GO:0005637 nuclear inner membrane	26	3.7E-4
		GO:0005762 mitochondrial large ribosomal subunit	18	9.2E-4
		GO:0005686 U2 snRNP	11	3.7E-3
		GO:0002189 ribose phosphate diphosphokinase complex	4	2.0E-2

	MF	GO:0004714 transmembrane receptor protein tyrosine kinase activity	84	9.4E-3
		GO:0004197 cysteine-type endopeptidase activity	27	1.7E-3
		GO:0003743 translation initiation factor activity	20	7.7E-4
		GO:0016493 C-C chemokine receptor activity	13	2.5E-5
		GO:0017056 structural constituent of nuclear pore	11	6.0E-3
	KEGG	hsa04658 Th17 cell differentiation	33	1.7E-3
		hsa04658 Th1 and Th2 cell differentiation	30	9.5E-4
		hsa00190 oxidative phosphorylation	29	2.0E-2
		hsa04672 intestinal immune network for IgA production	23	1.0E-5
		hsa04723 retrograde endocannabinoid signaling	22	9.0E-2
grey60	BP	GO:0006468 protein phosphorylation	96	4.9E-6
		GO:0060326 cell chemotaxis	29	3.6E-7
		GO:0030593 neutrophil chemotaxis	27	2.8E-5
		GO:0010572 positive regulation of platelet activation	10	1.5E-7
		GO:0002224 toll-like receptor signaling pathway	10	1.0E-2
	CC	GO:0000785 chromatin	198	1.1E-5
		GO:0000786 nucleosome	31	5.8E-3
		GO:0000307 cyclin-dependent protein kinase holoenzyme complex	14	4.6E-3
		GO:0008305 integrin complex	9	2.6E-2
		GO:1990779 glycoprotein Ib-IX-V complex	5	1.4E-3
	MF	GO:0004712 protein serine/threonine/tyrosine kinase activity	103	5.4E-7
		GO:0019957 C-C chemokine binding	13	3.6E-5
		GO:0008009 chemokine activity	13	4.6E-2
		GO:0003953 NAD+ nucleosidase activity	12	1.0E-3
		GO:0071723 lipopeptide binding	8	2.4E-4
	KEGG	hsa04658 Th17 cell differentiation	33	2.4E-3
		hsa04658 Th1 and Th2 cell differentiation	26	2.1E-2
		hsa04917 prolactin signaling pathway	26	3.6E-4
		hsa04911 insulin secretion	18	3.9E-2
		hsa04927 cortisol synthesis and secretion	17	1.2E-2
light green	BP	GO:0006468 protein phosphorylation	95	3.2E-5
		GO:0006935 chemotaxis	32	1.1E-3
		GO:0032543 mitochondrial translation	27	6.6E-4
		GO:0006913 nucleocytoplasmic transport	19	2.3E-4
		GO:0070106 interleukin-27-mediated signaling pathway	6	9.3E-4
	CC	GO:0005637 nuclear inner membrane	29	1.9E-5
		GO:0005762 mitochondrial large ribosomal subunit	18	9.3E-4
		GO:0005655 nucleolar ribonuclease P complex	5	1.5E-2

		GO:0042719 mitochondrial intermembrane space protein transporter complex	5	4.1E-3
		GO:0002199 zona pellucida receptor complex	4	8.4E-3
	MF	GO:0004712 protein serine/threonine/tyrosine kinase activity	85	7.2E-3
		GO:0003735 structural constituent of ribosome	33	2.2E-2
		GO:0016493 C-C chemokine receptor activity	14	3.7E-6
		GO:0017056 structural constituent of nuclear pore	11	6.2E-3
		GO:0001730	4	1.1E-2
	KEGG	hsa04658 Th17 cell differentiation	33	1.6E-3
		hsa04658 Th1 and Th2 cell differentiation	30	8.8E-4
		hsa04211 longevity regulating pathway	25	1.9E-2
		hsa04931 insulin resistance	22	3.6E-2
		hsa04723 retrograde endocannabinoid signaling	21	9.3E-3
light yellow	BP	GO:0006468 protein phosphorylation	90	3.3E-4
		GO:0000398 mRNA splicing, via spliceosome	46	8.7E-4
		GO:0006935 chemotaxis	32	1.0E-3
		GO:0032212 positive regulation of telomere maintenance via telomerase	15	1.0E-4
		GO:0006626 protein targeting to mitochondrion	13	4.4E-4
	CC	GO:0005637 nuclear inner membrane	26	3.5E-4
		GO:0005762 mitochondrial large ribosomal subunit	19	2.9E-4
		GO:0071005 U2-type precatalytic spliceosome	19	5.6E-5
		GO:0005832 chaperonin-containing T-complex	6	1.0E-2
		GO:0002199 zona pellucida receptor complex	4	8.3E-2
	MF	GO:0004712 protein serine/threonine/tyrosine kinase activity	84	8.8E-3
		GO:0003735 structural constituent of ribosome	34	1.6E-2
		GO:0003743 translation initiation factor activity	20	7.5E-4
		GO:0044183 protein folding chaperone	15	1.0E-2
		GO:0002189 ribose phosphate diphosphokinase complex	4	2.0E-2
	KEGG	hsa04658 Th17 cell differentiation	31	5.7E-3
		hsa04658 Th1 and Th2 cell differentiation	28	3.7E-3
		hsa05321 inflammatory bowel disease	18	5.5E-3
		map05320 autoimmune thyroid disease	14	1.3E-2
		hsa04911 insulin secretion	9	9.9E-3
magenta	BP	GO:0006357 regulation of transcription by RNA polymerase II	251	1.7E-2
		GO:0006468 protein phosphorylation	99	6.9E-7
		GO:0007229 integrin-mediated signaling pathway	33	2.2E-5
		GO:0060326 cell chemotaxis	26	1.5E-5
		GO:0002224 toll-like receptor signaling pathway	10	9.8E-3
	CC	GO:0000785 chromatin	188	3.0E-4

		GO:0000765 nucleosome	30	1.0E-2
		GO:0000307 cyclin-dependent protein kinase holoenzyme	15	1.5E-3
		GO:0008305 integrin complex	11	2.2E-3
		GO:1990779 glycoprotein Ib-IX-V complex	5	1.4E-3
	MF	GO:0004714 transmembrane receptor protein tyrosine kinase activity	99	4.4E-3
		GO:0004712 protein serine/threonine/tyrosine kinase activity	96	1.6E-6
		GO:0030527 structural constituent of chromatin	25	4.3E-3
		GO:0004197 cysteine-type endopeptidase activity	20	1.1E-2
		GO:0004714 transmembrane receptor protein tyrosine kinase activity	12	1.4E-2
	KEGG	hsa05225 hepatocellular carcinoma	51	1.2E-4
		hsa04935 growth hormone synthesis, secretion, and action	34	6.5E-3
		hsa04660 T cell receptor signaling pathway	31	3.8E-2
		hsa04720 long-term potentiation	18	8.3E-3
		hsa05332 graft-versus-host disease	17	1.7E-3
midnight blue	BP	GO:0006468 protein phosphorylation	92	1.3E-4
		GO:0060326 cell chemotaxis	21	3.6E-3
		GO:0006913 nucleocytoplasmic transport	20	6.3E-5
		GO:0051603 proteolysis involved in protein catabolic process	15	7.0E-5
		GO:0006164 purine nucleotide biosynthetic process	9	1.8E-3
	CC	GO:0005681 spliceosomal complex	38	2.1E-5
		GO:0005637 nuclear inner membrane	26	3.7E-4
		GO:0005762 mitochondrial large ribosomal subunit	18	9.2E-4
		GO:0042105 alpha-beta T cell receptor complex	6	1.6E-2
		GO:0005832 chaperonin-containing T-complex	6	1.1E-2
	MF	GO:0004712 protein serine/threonine/tyrosine kinase activity	82	1.8E-2
		GO:0004197 cysteine-type endopeptidase activity	28	7.4E-4
		GO:0003743 translation initiation factor activity	20	7.7E-4
		GO:0016493 C-C chemokine receptor activity	13	2.5E-5
		GO:0017056 structural constituent of nuclear pore	11	6.0E-3
	KEGG	map05322 systemic lupus erythematosus	24	6.4E-3
		T30312 glutathione metabolism	22	4.4E-4
		map04725 cholinergic synapse	19	7.3E-3
		hsa05321 inflammatory bowel disease	17	9.9E-3
		hsa05332 graft-versus-host disease	16	4.0E-3
red	BP	GO:0000398 mRNA splicing, via spliceosome	47	4.9E-4
		GO:0006935 chemotaxis	30	4.1E-3
		GO:0000079 regulation of cyclin-dependent protein serine/threonine kinase activity	30	4.5E-3

		GO:0032212 positive regulation of telomere maintenance via telomerase	25	3.0E-4
		GO:0006626 protein targeting to mitochondrion	13	2.4E-4
	CC	GO:0005681 spliceosomal complex	38	2.2E-5
		GO:0032543 mitochondrial translation	25	3.5E-4
		GO:0006913 nucleocytoplasmic transport	20	3.2E-4
		GO:0005832 chaperonin-containing T-complex	12	3.0E-2
		GO:0002199 zona pellucida receptor complex	4	3.3E-2
	MF	GO:0003735 structural constituent of ribosome	35	1.2E-3
		GO:0004197 cysteine-type endopeptidase activity	25	1.7E-3
		GO:0003743 translation initiation factor activity	20	7.7E-4
		GO:0030527 structural constituent of chromatin	15	6.6E-4
		GO:0008234 cysteine-type peptidase activity	14	3.9E-3
	KEGG	hsa04658 Th17 cell differentiation	33	1.3E-3
		hsa04658 Th1 and Th2 cell differentiation	30	7.4E-4
		hsa05321 inflammatory bowel disease	22	6.7E-4
		map05320 autoimmune thyroid disease	15	1.3E-3
		hsa04911 insulin secretion	10	8.5E-4
salmon	BP	GO:0006468 protein phosphorylation	92	1.2E-4
		GO:0006935 chemotaxis	35	1.1E-3
		GO:0032543 mitochondrial translation	30	6.6E-4
		GO:0006913 nucleocytoplasmic transport	19	2.3E-4
		GO:0070106 interleukin-27-mediated signaling pathway	6	9.3E-4
	CC	GO:0005681 spliceosomal complex	37	4.9E-5
		GO:0005637 nuclear inner membrane	25	6.7E-4
		GO:0005762 mitochondrial large ribosomal subunit	20	4.2E-4
		GO:0042105 alpha-beta T cell receptor complex	6	5.6E-3
		GO:0005832 chaperonin-containing T-complex	5	3.1E-3
	MF	GO:0004712 protein serine/threonine/tyrosine kinase activity	87	3.1E-3
		GO:0003735 structural constituent of ribosome	45	6.6E-3
		GO:0003743 translation initiation factor activity	37	3.5E-4
		GO:0044183 protein folding chaperone	22	7.4E-2
		GO:0002189 ribose phosphate diphosphokinase complex	8	5.2E-3
	KEGG	hsa04658 Th17 cell differentiation	33	5.7E-3
		hsa04658 Th1 and Th2 cell differentiation	30	3.7E-3
		hsa05321 inflammatory bowel disease	18	4.3E-2
		map05320 autoimmune thyroid disease	14	1.3E-3
		hsa04911 insulin secretion	9	1.4E-3
tan	BP	GO:0006468 protein phosphorylation	92	4.5E-4
		GO:0006935 chemotaxis	37	5.3E-3

		GO:0032543 mitochondrial translation	34	6.3E-4
		GO:0006913 nucleocytoplasmic transport	25	2.2E-4
		GO:0070106 interleukin-27-mediated signaling pathway	11	2.3E-4
	CC	GO:0000785 chromatin	174	5.0E-4
		GO:0000765 nucleosome	47	1.0E-2
		GO:0000307 cyclin-dependent protein kinase holoenzyme	18	1.5E-3
		GO:0008305 integrin complex	14	2.2E-3
		GO:1990779 glycoprotein Ib-IX-V complex	7	2.4E-3
	MF	GO:0004712 protein serine/threonine/tyrosine kinase activity	90	5.3E-3
		GO:0004197 cysteine-type endopeptidase activity	26	4.2E-4
		GO:0003743 translation initiation factor activity	23	3.5E-4
		GO:0016493 C-C chemokine receptor activity	15	2.2E-5
		GO:0017056 structural constituent of nuclear pore	15	2.3E-3
	KEGG	hsa04658 Th17 cell differentiation	33	8.1E-3
		hsa04658 Th1 and Th2 cell differentiation	30	3.4E-3
		hsa05321 inflammatory bowel disease	17	4.2E-2
		map05320 autoimmune thyroid disease	16	4.2E-3
		hsa04911 insulin secretion	15	2.2E-3
turquoise	BP	GO:0006357 regulation of transcription by RNA polymerase II	251	3.7E-4
		GO:0006468 protein phosphorylation	94	1.9E-7
		GO:0007229 integrin-mediated signaling pathway	36	2.2E-5
		GO:0060326 cell chemotaxis	24	1.7E-5
		GO:0002224 toll-like receptor signaling pathway	15	2.8E-3
	CC	GO:0000785 chromatin	188	6.8E-3
		GO:0000765 nucleosome	35	1.0E-2
		GO:0000307 cyclin-dependent protein kinase holoenzyme	17	5.5E-3
		GO:0008305 integrin complex	15	3.2E-3
		GO:1990779 glycoprotein Ib-IX-V complex	7	6.4E-3
	MF	GO:0004714 transmembrane receptor protein tyrosine kinase activity	88	4.6E-3
		GO:0004197 cysteine-type endopeptidase activity	29	2.2E-4
		GO:0030527 structural constituent of chromatin	28	4.3E-3
		GO:0004197 cysteine-type endopeptidase activity	25	3.1E-3
		GO:0004714 transmembrane receptor protein tyrosine kinase activity	13	3.4E-3
	KEGG	hsa04658 Th17 cell differentiation	33	6.6E-4
		hsa04658 Th1 and Th2 cell differentiation	30	5.8E-4
		hsa04211 longevity regulating pathway	26	1.9E-2
		hsa04931 insulin resistance	25	3.3E-4
		hsa04723 retrograde endocannabinoid signaling	23	2.3E-3

Table S2. List of overlapping hub genes from each module obtained from the three algorithms.

Module	Hub Genes
brown	CD44, BRCA1, TLR4, ITGAM, CD8A, CCL5, TP53, MED1, STAT1, CCNB1, CD4, MYC, JUN, CASP3, CCNA42
cyan	SYK, KIT, CD44, BRCA1, TLR4, ITGAM, FOS, MAPK1, CXCR4, BCL2, CCL2, MAPK14, PRKCA, CD36, FGF2, STAT1, ITGB2, EP300, TLR2, CXCL8, TGFB11, CD4, IL1B, ICAM1, GNB1, CSF1R, MAPK3, APOE, MMP9, PTPRC
dark turquoise	KIT, HSP90AB1, BRCA1, IFNG, PIK3R1, JAK2, GSK3B, CALML6,, CD8A, CCL2, CDC20, EP300, SMAD3, CCNB1, AURKA, CTNNB1, TGFB1, TOP2A, EGF, FYN, CDK2, JUN, CCNA2, EZH2, PTPRC
green yellow	HSP90AB1, MRPL3, IFNG, CD8A, RPL35, BCL2, TP53, MED1, STAT1, EP300, CD4, RPS5, CCNA2, RPS24
grey60	CD44, BRCA1, TLR4, ITGAM, FOS, DDX58, JAK2, CALML6, MAPK1, CXCR4, CCR5, BCL2, CCL2, MAPK14, CCL5, PTEN, STAT1, ITGB2, EP300, CALML5, TLR2, CXCL8, EGF, CD4, IL1B, ICAM1, CSF1R, JUN, CASP3, MAPK3, MMP9, PTPRC
light green	HSP90AB1, IFNG, CD8A, RPL35, BCL2, TP53, MED1, STAT1, EP300, CCNB1, CTNNB1, CD4, RPS5, CCNA2, RPS24
light yellow	HSP90AB1, IFNG, CD8A, RPL35, TP53, MED1, STAT1, EP300, CCNB1, CTNNB1, CD4, RPS5, CCNA2, RPS24
magenta	CD44, BRCA1, TLR4, ITGAM, FOS, JAK2, CALML6, MAPK1, CXCR4, BCL2, CCL2, MAPK14, PTEN, STAT1, ITGB2, EP300, CALML5, TLR2, CXCL8, CTNNB1, CD4, IL1B, ICAM1, JUN, CD40, CASP3, MAPK3, MMP9, PTPRC
midnight blue	HSP90AB1, MRPL3, IFNG, CD8A, RPL35, TP53, MED1, STAT1, EP300, IMP3, CTNNB1, CD4, RPS5, RPS24
red	HSP90AB1, IFNG, CD8A, RPL35, TP53, MED1, STAT1, IMP3, CTNNB1, CD4, RPS5, CCNA2, RPS24
salmon	IFNG, CD8A, MED1, EP300, CTNNB1, CD4, CCNA2
tan	HSP90AB1, IFNG, CD8A, RPL35, BCL2, TP53, MED1, STAT1, EP300, CCNB1, CTNNB1, CD4, MYC, RPS5, CCNA2, RPS24
turquoise	HSP90AB1, IFNG, CD8A, RPL35, BCL2, TP53, MED1, STAT1, EP300, CCNB1, CTNNB1, CD4, MYC, RPS5, CCNA2, RPS24

Table S3. List of alternatively spliced isoforms found to participate in T1D, PSR, SSc, and SLE susceptibility.

Disease	Gene/Isoform	Consequence	Ref.
T1D	G6PC2	Differentially expresses in thymus and pancreas and induces auto-tolerance	[1,2]
	Bim/Bim S	Stimulates apoptotic activity	[3,4]
	Adora1/Adora1-Var	Inhibits Adora1	[5,6]
	TAP2	Presents more antigens	[7]
	IA-2/IA-2Δ13, Δ14	Synthesizes new antigenic epitopes	[8]
	FOXP3/Foxp3-E2	Influences T_{reg} cell function	[9,10]
	Deaf1/Deaf1-Var1	Initiates under-expression of peripheral tissue antigens in lymph nodes	[11,12]
	CTLA4/sCTLA4	Enhances T_{reg} cell function	[13,14]
PSR	KLK10	Improves apoptotic activity	[15,16]
	IL36RN	Initiates IL-36Ra synthesis	[17,18]
	CARD14	Generates protein variants that influence NF-κB activation	[19,20]
	TRAF3IP2	Alters IL-17 signaling	[21]
	Fibronectin isoforms	Involves in sensitization of keratinocytes to mitogenic signals	[22]
SSc	VEGF/VEGF 165b	Primes angiogenesis inefficiency	[23,24]
	CTLA4/sCTLA4	Interferes with B7 and blocks negative signals	[25]
	IL-4/ IL-4δ2	Inhibits IL-4	[26,27]
SLE	CD72/ CD72Δex8	Inhibits BCR signaling	[28,29]
	IRF5/V1-11	Initiates overexpression of IRF5 and IFN	[30]
	BANK1/Δ2	Inefficiently transmits downstream signaling	[30]
	CTLA4/sCTLA4	Interferes with B7 and blocks negative signals	[13,14]
	TCRζ	Disrupts T_{reg}-T_{H17} cell balance	[31,32]
	RasGRP1	Inhibits T cell maturation	[33,34]

References

1. Overway, E.M.; Bosma, K.J.; Claxton, D.P.; Oeser, J.K.; Singh, K.; Breidenbach, L.B.; McHaourab, H.S.; Davis, L.K.; OBrien, R.M. Nonsynonymous single-nucleotide polymorphisms in the G6PC2 gene affect protein expression, enzyme activity, and fasting blood glucose. *J. Biological Chemistry* **2022**, *298*, 101534. https://doi.org/10.1016/J.JBC.2021.101534.
2. De Jong, V.M.; Abreu, J.R.F.; Verrijn Stuart, A.A.; Van Der Slik, A.R.; Verhaeghen, K.; Engelse, M.A.; Blom, B.; Staal, F.J.T.; Gorus, F.K.; Roep, B.O. Alternative splicing and differential expression of the islet autoantigen IGRP between pancreas and thymus contributes to immunogenicity of pancreatic islets but not diabetogenicity in humans. *Diabetologia* **2013**, *56*, 2651–2658. https://doi.org/10.1007/S00125-013-3034-6.
3. Nogueira, T.C.; Paula, F.M.; Villate, O.; Colli, M.L.; Moura, R.F.; Cunha, D.A.; Marselli, L.; Marchetti, P.; Cnop, M.; Julier, C.; et al. GLIS3, a susceptibility gene for type 1 and type 2 diabetes, modulates pancreatic beta cell apoptosis via regulation of a splice variant of the BH3-only protein Bim. *PLoS Genet* **2013**, *9*, e1003532. https://doi.org/10.1371/JOURNAL.PGEN.1003532.
4. Zhang, Q.; Li, G.; Kong, J.; Dai, J.; Fan, Z.; Li, J. miR-222-3p reduces neuronal cell apoptosis and alleviates spinal cord injury by inhibiting Bbc3 and Bim. *Neurosci. Res.* **2023**, *188*, 39–50. https://doi.org/10.1016/J.NEURES.2022.10.008.
5. Yip, L.; Taylor, C.; Whiting, C.C.; Fathman, C.G. Diminished adenosine a1 receptor expression in pancreatic a-cells may contribute to the patholog y of type 1 diabetes. *Diabetes* **2013**, *62*, 4208–4219. https://doi.org/10.2337/DB13-0614/-/DC1.
6. Beach, K.M.; Hung, L.F.; Arumugam, B.; Smith, E.L.; Ostrin, L.A. Adenosine receptor distribution in Rhesus monkey ocular tissue. *Exp. Eye Res.* **2018**, *174*, 40–50. https://doi.org/10.1016/J.EXER.2018.05.020.
7. Wiśniewski, A.; Wilczyńska, K.; Wagner, M.; Jasek, M.; Niepiekło-Miniewska, W.; Nowak, I.; Matusiak, Ł.; Szczerkowska-Dobosz, A.; Kuśnierczyk, P. Is the TAP2 single nucleotide polymorphism rs241447 truly associated with psoriasis in Poles? *Hum. Immunol.* **2020**, *81*, 85–90. https://doi.org/10.1016/J.HUMIMM.2020.01.005.
8. Dai, Z.X.; Zhang, G.H.; Zhang, X.H.; Zheng, Y.T. Identification and characterization of a novel splice variant of rhesus macaque MHC IA. *Mol. Immunol.* **2013**, *53*, 206–213. https://doi.org/10.1016/J.MOLIMM.2012.08.006.
9. De Rosa, V.; Galgani, M.; Porcellini, A.; Colamatteo, A.; Santopaolo, M.; Zuchegna, C.; Romano, A.; De Simone, S.; Procaccini, C.; La Rocca, C.; et al. Glycolysis controls the induction of human regulatory T cells by modulating the expression of FOXP3 exon 2 splicing variants. *Nat. Immunol.* **2015**, *16*, 1174–1184. https://doi.org/10.1038/NI.3269.
10. Cassidy, M.F.; Herbert, Z.T.; Moulton, V.R. Splicing factor SRSF1 controls autoimmune-related molecular pathways in regulatory T cells distinct from FoxP3. *Mol. Immunol.* **2022**, *152*, 140–152. https://doi.org/10.1016/J.MOLIMM.2022.10.017.
11. Chen, S.; Deng, X.; Xiong, J.; He, F.; Yang, L.; Chen, B.; Chen, C.; Zhang, C.; Yang, L.; Peng, J.; et al. De novo variants of DEAF1 cause intellectual disability in six Chinese patients. *Clinica Chimica Acta* **2021**, *518*, 17–21. https://doi.org/10.1016/J.CCA.2021.02.026.
12. Yip, L.; Creusot, R.; Su, L.; Fathman, C. Inflammation-induced Changes in Deaf1 Splicing Alter Peripheral Tissue Antigen Gene Expression in the Pancreatic Lymph Node during the Pathogenesis of Type I Diabetes. *Clin. Immunol.* **2010**, *135*, S72. https://doi.org/10.1016/J.CLIM.2010.03.220.
13. Gerold, K.D.; Zheng, P.; Rainbow, D.B.; Zernecke, A.; Wicker, L.S.; Kissler, S. The soluble CTLA-4 splice variant protects from type 1 diabetes and potentiates regulatory T-cell function. *Diabetes* **2011**, *60*, 1955–1963. https://doi.org/10.2337/DB11-0130.
14. Rasmussen, T.A.; Zerbato, J.M.; Rhodes, A.; Tumpach, C.; Dantanarayana, A.; McMahon, J.H.; Lau, J.S.Y.; Chang, J.J.; Gubser, C.; Brown, W.; et al. Memory CD4+ T cells that co-express PD1 and CTLA4 have reduced response to activating stimuli facilitating HIV latency. *Cell Rep. Med.* **2022**, *3*, 100766. https://doi.org/10.1016/J.XCRM.2022.100766.
15. Alexopoulou, D.K.; Papadopoulos, I.N.; Scorilas, A. Clinical significance of kallikrein-related peptidase (KLK10) mRNA expression in colorectal cancer. *Clin. Biochem.* **2013**, *46*, 1453–1461. https://doi.org/10.1016/J.CLINBIOCHEM.2013.03.002.
16. Adamopoulos, P.G.; Kontos, C.K.; Scorilas, A. Identification and molecular cloning of novel transcripts of the human kallikrein-related peptidase 10 (KLK10) gene using next-generation sequencing. *Biochem. Biophys. Res. Commun.* **2017**, *487*, 776–781. https://doi.org/10.1016/J.BBRC.2017.04.078.
17. Onoufriadis, A.; Simpson, M.A.; Pink, A.E.; Di Meglio, P.; Smith, C.H.; Pullabhatla, V.; Knight, J.; Spain, S.L.; Nestle, F.O.; Burden, A.D.; et al. Mutations in IL36RN/IL1F5 Are Associated with the Severe Episodic

Inflammatory Skin Disease Known as Generalized Pustular Psoriasis. *Am. J. Hum. Genet.* **2011**, *89*, 432–437. https://doi.org/10.1016/J.AJHG.2011.07.022.
18. Mizukawa, I.; Kamata, M.; Uchida, H. 32339 Expression of interleukin 36 receptor antagonist in a patient with generalized pustular psoriasis harboring the p.Pro82Leu variant in the IL36RN gene. *J. Am. Acad. Dermatol.* **2022**, *87*, AB59. https://doi.org/10.1016/J.JAAD.2022.06.270.
19. Rossel, V.; Baniel, A.; Wertheim-Tysarowska, K.; Seyger, M.; Spruijt, L.; Bekkenk, M.; Vreeburg, M.; Sprecher, E.; Steijlen, P.; van Geel, M.; et al. 819 International cohort of 19 patients with CARD14-associated papulosquamous eruption: The quest for a genotype-phenotype correlation and successful therapeutic intervention. *J. Investig. Dermatol.* **2023**, *143*, S141. https://doi.org/10.1016/J.JID.2023.03.829.
20. Mellett, M. Regulation and dysregulation of CARD14 signalling and its physiological consequences in inflammatory skin disease. *Cell. Immunol.* **2020**, *354*, 104147. https://doi.org/10.1016/J.CELLIMM.2020.104147.
21. Wu, B.; Gong, J.; Yuan, S.; Zhang, Y.; Wei, T. Patterns of evolutionary selection pressure in the immune signaling protein TRAF3IP2 in mammals. *Gene* **2013**, *531*, 403–410. https://doi.org/10.1016/J.GENE.2013.08.074.
22. Otten, A.B.C.; Amarbayar, O.; Cai, P.; Cheng, B.; Qu, K.; Sun, B.K. The Long Noncoding RNA PRANCR Is Associated with Alternative Splicing of Fibronectin-1 in Keratinocytes. *J. Investig. Dermatol.* **2023**, *143*, 1825–1830.e6. https://doi.org/10.1016/J.JID.2023.01.038.
23. Li, Q.; Zeng, C.; Liu, H.; Yung, K.W.Y.; Chen, C.; Xie, Q.; Zhang, Y.; Wan, S.W.C.; Mak, B.S.W.; Xia, J.; et al. Protein-protein interaction inhibitor of SRPKs alters the splicing isoforms of VEGF and inhibits angiogenesis. *IScience* **2021**, *24*, 102423. https://doi.org/10.1016/J.ISCI.2021.102423.
24. Jiao, L.; Gong, M.; Yang, X.; Li, M.; Shao, Y.; Wang, Y.; Li, H.; Yu, Q.; Sun, L.; Xuan, L.; et al. NAD+ attenuates cardiac injury after myocardial infarction in diabetic mice through regulating alternative splicing of VEGF in macrophages. *Vascul. Pharmacol.* **2022**, *147*, 107126. https://doi.org/10.1016/J.VPH.2022.107126.
25. AlFadhli, S.; Nizam, R. Differential expression of alternative splice variants of CTLA4 in Kuwaiti autoimmune disease patients. *Gene* **2014**, *534*, 307–312. https://doi.org/10.1016/J.GENE.2013.10.034.
26. Luzina, I.G.; Keegan, A.D.; Heller, N.M.; Rook, G.A.W.; Shea-Donohue, T.; Atamas, S.P. Regulation of inflammation by interleukin-4: A review of "alternatives". *J. Leukoc. Biol.* **2012**, *92*, 753–764. https://doi.org/10.1189/JLB.0412214.
27. Diogo, G.R.; Sparrow, A.; Paul, M.J.; Copland, A.; Hart, P.J.; Stelter, S.; van Dolleweerd, C.; Drake, P.M.W.; Macallan, D.C.; Reljic, R. Murine IL-4Δ2 splice variant down-regulates IL-4 activities independently of IL-4Rα binding and STAT-6 phosphorylation. *Cytokine* **2017**, *99*, 154–162. https://doi.org/10.1016/J.CYTO.2017.09.007.
28. Bie Petersen, C.; Nygård, A.B.; Fredholm, M.; Aasted, B.; Salomonsen, J. Various domains of the B-cell regulatory molecule CD72 has diverged at different rates in mammals: Cloning, transcription and mapping of porcine CD72. *Dev. Comp. Immunol.* **2007**, *31*, 530–538. https://doi.org/10.1016/J.DCI.2006.07.008.
29. Hitomi, Y.; Adachi, T.; Tsuchiya, N.; Honda, Z.I.; Tokunaga, K.; Tsubata, T. Human CD72 splicing isoform responsible for resistance to systemic lupus erythematosus regulates serum immunoglobulin level and is localized in endoplasmic reticulum. *BMC Immunol.* **2012**, *13*, 72. https://doi.org/10.1186/1471-2172-13-72.
30. Stone, R.C.; Du, P.; Feng, D.; Dhawan, K.; Rönnblom, L.; Eloranta, M.L.; Donnelly, R.; Barnes, B.J. RNA-Seq for enrichment and analysis of IRF5 transcript expression in SLE. *PLoS ONE* **2013**, *8*, e54487. https://doi.org/10.1371/JOURNAL.PONE.0054487.
31. Zha, X.; Yan, X.; Shen, Q.; Wu, X.; Chen, S.; Li, B.; Yang, L.; Li, Y. Dysexpression of TCRζ Related Genes in the Patients with Chronic Myeloid Leukemia. *Blood* **2012**, *120*, 4832. https://doi.org/10.1182/BLOOD.V120.21.4832.4832.
32. Raab, M.; Da Silva, A.J.; Findell, P.R.; Rudd, C.E. Regulation of Vav–SLP-76 Binding by ZAP-70 and Its Relevance to TCRζ/CD3 Induction of Interleukin-2. *Immunity* **1997**, *6*, 155–164. https://doi.org/10.1016/S1074-7613(00)80422-7.
33. Huang, H.; Jin, T.; Wang, L.; Wang, F.; Zhang, R.; Pan, Y.; Wang, Z.; Chen, Y. The RAS Guanyl Nucleotide-releasing Protein RasGRP1 Is Involved in Lymphatic Development in Zebrafish. *J. Biol. Chem.* **2013**, *288*, 2355–2364. https://doi.org/10.1074/JBC.M112.418202.
34. Tazmini, G.; Beaulieu, N.; Woo, A.; Zahedi, B.; Goulding, R.E.; Kay, R.J. Membrane localization of RasGRP1 is controlled by an EF-hand, and by the GEF domain. *Biochim. Et Biophys. Acta (BBA)-Mol. Cell Res.* **2009**, *1793*, 447–461. https://doi.org/10.1016/J.BBAMCR.2008.12.019.
